# How safe is it to plan a vaginal breech birth with OptiBreech collaborative care?: analysis of cumulative data within the OptiBreech Multiple Trials Cohort

**DOI:** 10.3310/nihropenres.13500.1

**Published:** 2023-11-23

**Authors:** Shawn Walker, Sabrina Das, Kate Stringer, Emma Spillane, Amy Meadowcroft, Siân Davies, Jacana Bresson, Alice Hodder, Jasmine Kang, The OptiBreech Collaborative

**Affiliations:** 1 King's College London, London, England, UK; 2 Imperial College Health Partners, London, England, UK; 3 Imperial College London, London, England, UK; 4 Surrey and Sussex Healthcare NHS Trust, Redhill, England, UK; 5 Kingston Hospital NHS Foundation Trust, London, England, UK; 6 Northern Care Alliance NHS Foundation Trust, Salford, England, UK

**Keywords:** breech presentation, physiological breech birth, breech specialist midwives, OptiBreech, interim analysis, cohort study, multi-trial cohort

## Abstract

**Background:**

OptiBreech collaborative care is a multi-disciplinary care pathway for breech presentation at term. The OptiBreech Multiple Trial Cohort is designed to host multiple trials related to care for breech presentation. This design enables prospective data collection for a large cohort of women planning a vaginal breech birth (VBB), to assess rare safety outcomes, while answering questions requiring a smaller, randomised sample nested within this cohort.

**Methods:**

OptiBreech database currently contains participants recruited from 10 January 2022, including 67 women randomised to either OptiBreech care or standard care, and 116 women who received OptiBreech care and were not randomised. Primary outcomes included vaginal birth rate, composite neonatal morbidity and mortality and composite maternal neonatal morbidity and mortality. Descriptive statistics for the entire cohort were analysed in SPSS Version 29. Sub-group analyses were identified through participant involvement and engagement work as important to support informed decision-making.

**Results:**

Of 97 women who planned a VBB at any point, 44 (45.4%) achieved a vaginal birth, compared to 29/77 (37.7%) of women who did not plan a VBB. Admission rates to a neonatal unit were similar, 4/97 (4.1%) versus 3/77 (3.9%). In this cohort, there was no severe neonatal morbidity following planned VBB, compared to 3/77 (3.9%) among the cohort who did not plan a vaginal breech birth and 2/52 (3.7%) among women who planned a cephalic birth. Severe maternal morbidity following planned VBB was 7/89 (7.9%), compared to 9/76 (11.8%) for women who did not plan a VBB and 8/54 (14.8%) for women who planned a cephalic birth.

**Conclusions:**

Planning a VBB with OptiBreech collaborative care has thus far been as safe as not planning a VBB. Detecting differences in rare outcomes will require thousands of births. Outcomes will continue to be monitored and reported here as the cohort grows.

## Plain English Summary

Around 1 in 20 pregnant women / birthing people experience a breech presentation (where baby is bottom-first instead of head-down in the womb) after 35 weeks of pregnancy. Women with a breech baby can choose to birth vaginally or by caesarean. Both breech babies and their mothers are at a higher risk of poor outcomes regardless of their type of birth, compared to pregnancies where babies have not been breech. It can also be difficult for clinicians to gain experience, hence limiting options and increasing risks for unexpected breech births.

OptiBreech collaborative care is a new care model that aims to improve safety and care to offer women real choice. The need for this research, how it was designed, and how the on-going results are interpreted has been informed by women who use NHS services.

It will require thousands of births, and take a long time, to determine if OptiBreech care reduces the number of babies who are seriously ill or die around the time of birth. This is already a low number, but we hope to reduce it further. We will also consider how women are able to give birth and their health after birth.

It is important to women and clinicians that we share results of all births within our database regularly, so that they can make an informed decision about whether they would like to participate in OptiBreech research. Sponsors, funders, and ethics committees that oversee our research would like this information to make decisions about whether the research should continue.

This research will eventually help policy makers decide if OptiBreech care should be implemented as standard within all NHS services. We will work with our patient and public involvement group to share findings through health professional training, conferences, reports to funders, stakeholder events, and social media.

## Introduction

OptiBreech collaborative care is a specialist, multi-disciplinary care pathway for women and birthing people with a breech-presenting fetus at term, developed out of previous research and in collaboration with service users and clinicians ^1– 4^.

Approximately 1 in 20 pregnant women have a breech presenting fetus at the end of pregnancy. Babies are at higher risk of poor outcomes, regardless of the mode of birth ^5^. Mothers experience increased rates of surgical delivery and birth trauma. Balancing these risks through person-centred care, centred on the person’s values and life context, is improved when obstetricians and midwives have skill and experience in vaginal breech birth (VBB). Due to the way services are currently delivered in standard services, experience is difficult to acquire.

Variations in skill and experience are dangerous and expensive. In 2021–22, the National Health Services’ (NHS) financial liabilities for claims of obstetric negligence causing cerebral palsy was £36.8 billion ^6^. Twelve percent of obstetrics claims for cerebral palsy relate to poorly managed VBBs, despite representing only 0.3% of total births ^7^. Our team developed an evidence-based management algorithm that aims to reduce the leading cause of breech birth-related injury: asphyxia ^8– 10^.

Current NHS strategies to reduce risk focus on reducing the numbers of VBBs, through external cephalic version (ECV, turning the baby head-down) and/or planned caesarean birth (CB) ^11^. An unintended effect has been decline in health care professionals’ skills ^12^. This strategy has reduced the VBBs we can anticipate, resulting in a lack of learning opportunities for staff to safely manage those we cannot predict. Due to maternal choice, lack of universal diagnosis by ultrasound scan ^13^, and late-changing fetal positions (unstable lie), VBBs continue to occur, but their rarity makes them vulnerable.

OptiBreech collaborative care is an alternative strategy. The strategy is to support women who choose to plan a VBB, prepare for these births and manage them according to our algorithm. Professionals with advanced training in physiological breech birth ^14, 15^ attend VBBs whenever possible. The service is co-ordinated by a Breech Specialist Midwife ^1, 4^ with support from a Breech Lead Obstetrician. A small, experienced team provides continuity for women and professionals ^2^; their role is to train and support the wider team. This practice includes the option of upright maternal birthing positions ^4, 16^.

Within the OptiBreech collaborative care pathway, women are offered all guideline-recommended options: VBB, ECV, and planned CB. Women find this service beneficial, regardless of their mode of birth ^1^. Due to substantial differences in clinical practices and the way services are delivered, OptiBreech care is expected to result in different safety outcomes from standard care. The current logic model and TIDieR checklist ^17^ for OptiBreech collaborative care have been reported in previous publications ^1, 18^. The OptiBreech training package has been described in detail in evaluation publications ^14, 15^. The OptiBreech Clinical Practice Guideline is included in the protocol uploaded to the ISRCTN registration ^19^. Much of this information is also available on the project’s engagement website, optibreech.uk.

Patient and Public Involvement and Engagement (PPIE) work aimed to ensure the study’s design, analysis, reporting, and interpretation were influenced by women who have lived experience of planning or attempting to plan a VBB. Previous research indicated this population was least well served within current NHS standard care, and this work aimed to improve their outcomes and experience of care ^1, 20– 22^, while maintaining choice and good outcomes for all women using the service.

This paper reports an interim analysis of all data available within the OptiBreech database to 8 September 2023. The purpose of reporting this analysis is to provide women and clinicians with as much up-to-date information as possible about what they can expect when planning a VBB with OptiBreech care. We have chosen this publication venue (NIHR Open Research) to enable transparent reporting and updating as the cohort grows, so that all stakeholders have contemporaneous information on which to base decisions.

## Methods

### Study design

The OptiBreech Multiple Trial Cohort was designed to host multiple trials related to care for breech presentation in the third trimester and birth. This is to enable prospective data collection for a large cohort of women planning a VBB with OptiBreech collaborative care, to assess rare safety outcomes, while answering questions requiring a smaller, randomised sample that could be nested within this cohort.

### Patient and Public Involvement

Stakeholder involvement was facilitated through multiple public meetings, held in person and on-line during the research design stage ^23^. Two lay members of the Trial Steering Committee and one member of the research team were service users with lived experience of planning a VBB. Additional service users with lived experience of planning a VBB participated as members of the research team during qualitative work to refine the OptiBreech care pathway intervention ^1, 22^ and consensus work to identify and prioritise outcome measures ^24, 25^.

PPIE input especially influenced the way we analysed and presented our results. Our PPIE group prioritised knowing how outcomes for VBBs compared with those for planned and actual cephalic births, regarded as the ‘normal’ care pathway. One of the challenges in breech research is the constantly moving parts – fetal presentation can change up to the point of labour and sometimes during; women may initially prefer one plan but change their minds for various reasons other than clinical concerns; and for each planned mode of birth, a portion of women will have a different mode of birth than the one they chose, due to the unpredictable nature of labour and birth.

For this reason, we have performed several subgroup analyses that interrogate the data from different points of view. For each section, we begin with the question that the analysis answers, from the point of view of policy makers and/or service leaders, and from the point of view of women and birthing people making informed decisions about their care options. Service users also advocated for an equity analysis due to growing awareness of increased risk of adverse outcomes among women of minoritised ethnicity and skin colour in the UK ^26^. Demographic information has been reported in line with the latest NICE style guide ^27.^

### Inclusion and exclusion criteria

Inclusion criteria for the OptiBreech cohort are:

- Live, singleton pregnancy with a breech-presenting fetus confirmed by ultrasound scan;
- Over 16 years of age;
- Referred for specialist care for breech presentation antenatally from 32 weeks;
- Breech presentation from 37 weeks discovered in labour;
- Requesting or preferring a vaginal birth; and
- Giving informed consent to participate to contribute data to the cohort study.

Exclusion criteria for the cohort are:

- Absolute reason for caesarean section already exists (eg. placenta praevia major);
- Requesting a caesarean section prior to recruitment;
- Multiple pregnancy;
- Life-threatening congenital anomaly; or
- Not consenting to contribute data to the cohort study

### Currently included studies

**Figure 1.**
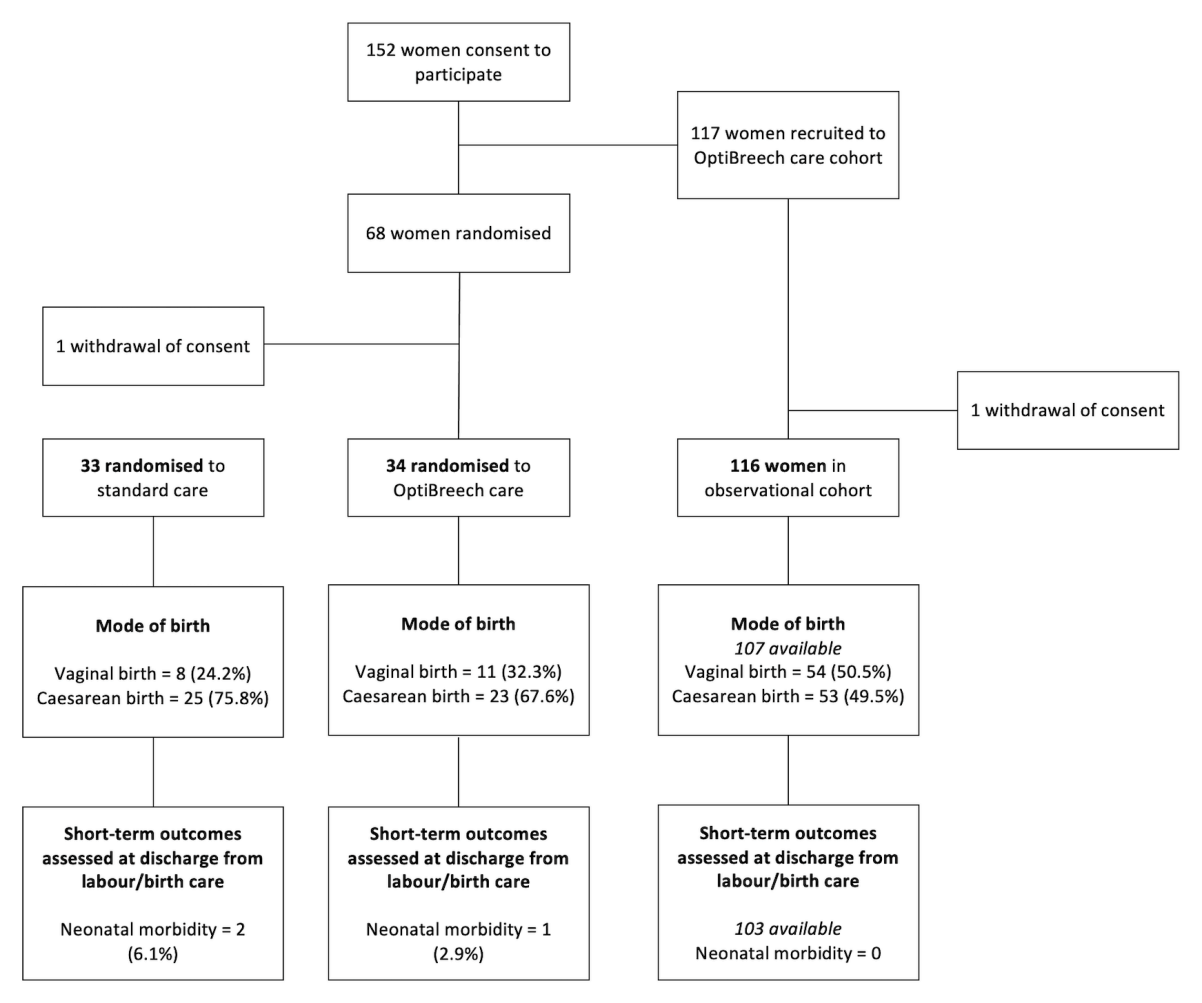
Participant Flow.

Studies currently included in the OptiBreech database are listed below and summarised in Figure 1: Participant Flow

1) *The OptiBreech Care Trial: a feasibility study for a pragmatic trial of care for women with a breech-presenting baby at term*. Randomised participants (68) were recruited between 10 January 2022 and 09 June 2022. Inclusion and exclusion criteria were more narrowly defined and are reported in detail in the pilot trial report ^28^. Cohort participants were recruited until 8 September 2023 and focused on women who preferred to plan a VBB regardless of whether their baby remained in a breech position. This study was funded by the UK National Institute for Health and Care Research (NIHR, 300582) and sponsored by King’s College London. Ethics approval was obtained from the West London & GTAC Research Ethics Committee (21/LO/0808, 19 November 2021). The pilot trial was prospectively registered with the ISRCTN (14521381, 18 October 2021) ^19^. The full protocol is available on the trial registration page ^19^.

### Consent process

Participants were recruited following a referral for counselling and/or care relating to breech presentation in the third trimester. The consent and randomisation process for the OptiBreech Care feasibility pilot has been described elsewhere ^28^. Information sources about the OptiBreech Care pathway, including all Participant Information Sheets and Consent Forms, was available via the OptiBreech engagement website (optibreech.uk) and wherever possible provided to women in advance of their appointment. Participants randomised to or requesting OptiBreech care received collaborative care co-ordinated and led by a specialist midwife. They were informed that this was a new care pathway and that they could request standard obstetric care if they preferred. Each participant provided consent to participate via written or e-consent form. Demographic data on ethnicity and gender was self-reported at the same time. Following the end of the pilot trial, recruitment focused on women requesting OptiBreech care due to a preference for vaginal birth, regardless of fetal position.

Participants were able to withdraw consent at any time if they wished. Where consent was withdrawn, this is indicated in the Participant Flow ( Figure 1), but no data has been reported.

### Procedures

Participants in the ‘standard care’ arm have received care that does not involve access to OptiBreech-trained specialists for antenatal care or birth. Generally, they were offered ECV as a first-line intervention and/or referred to their named obstetric consultant’s antenatal clinic for further counselling regarding mode of birth if declined. Participants in the OptiBreech care arm were counselled by a member of the OptiBreech team and were offered the option of planning a VBB with OptiBreech support, attempting an ECV, or planning a pre-labour CB from 39 weeks gestation.

### Outcomes

This report (v1.0) includes the cohort’s demographics and short-term mode of birth, safety, infant feeding and fidelity outcomes, measured at discharge from birth care. Below each table, a key is included that defines medical terms and abbreviations and provides information on how and when the outcomes included in that table were measured.

### Statistical analysis

Descriptive statistics only are reported. The study was not designed or powered to enable any inferential statistics. However, one aim of reporting the descriptive data in this way is to enable the design of future, appropriately powered investigations, based on the descriptive incidence rates.

This report is an interim analysis of a live, accumulating data set. We deal with missing data by reporting the current denominator of available data, for each outcome, where this is not equivalent to the total number of cases. During each interim analysis process, the team cleans the data by identifying missing data points and following up with site PIs, to ensure they are available for future cohort analyses.

Analysis was initially done by one member of the team (SW or SMD) using SPSS Version 29.0.1.0. Table data were checked against output files by other members of the team (SW, SMD, or JB). To verify the analysis, JK and AH repeated a sub-section of the analysis by randomisation arm / cohort using Stata/MP version 17.0.

Baseline demographics are analysed by cohort (randomisation arm, non-randomised cohort, and overall OptiBreech care cohort).

Presentation on admission, mode of birth and safety outcomes were assessed by: 1) cohort; 2) plan following first counselling (intention to treat); 3) those who planned a VBB at any point versus those who had not and those who had planned a cephalic birth; 4) actual VBBs versus vaginal cephalic births; 5) presentation on admission for labour/birth care; and 6) ethnicity. The group ‘did not plan a VBB at any point’ includes women who planned a caesarean birth and women who planned a cephalic birth, including those whose fetus was discovered to be presenting breech in labour. ‘Planned cephalic births’ included all those whose breech presentations were identified in the third trimester whose baby turned spontaneously or via ECV following recruitment, and those whose fetus was discovered to be presenting breech in labour but had planned a cephalic birth.

These subgroup analyses were chosen because 1) this comparison enables comparison of the non-randomised cohort with the randomised cohorts; 2) PPI group members valued this analysis to support informed decision-making; 3) these are the comparisons most often used in large observational cohort studies; 4) this comparison was prioritised by PPI group members; 5) cephalic presentation at birth is evaluated as an outcome in Cochrane Reviews concerning the management of breech presentation at term; and 6) our PPI group was keen to ensure non-white-British and Black or Brown participants were not disadvantaged within this model of care.

Infant feeding outcomes are analysed by cohort (standard care vs OptiBreech care) and whether the women had planned a VBB or cephalic birth.

Fidelity measures are reported for actual VBBs only, analysed according to whether there was a professional present who had completed the OptiBreech training and/or met all proficiency criteria, versus whether there was no OptiBreech trained professional present.

### Role of the funding source

SW and this research are funded by a National Institute for Health and Care Research (NIHR) Advanced Fellowship. The NIHR had no role in study design, data collection, data analysis, data interpretation, or writing of the report. Breech Birth Network, a not-for-profit Community Interest Company, provided training for OptiBreech teams, educational resources, and funding for PPIE, and conference presentations.

## Results

Results are presented by sub-group analysis category. With each table, we include the key questions the analysis seeks to answer, by stakeholder group. In all instances, it is not yet possible to detect any significant differences between groups, due to the small sample size.

### Analysis by randomisation group / cohort

**Table 1 Key Questions:**

1) How do characteristics of participants differ between ‘standard care’ and ‘OptiBreech care’ groups?
2) Are people from minoritized groups able to access participation in this research?

**Table 1.** Baseline characteristics, by randomisation group / cohort.

Outcome	Randomised Standard Care (nested RCT)	Randomised OptiBreech Care (nested RCT)	Cohort OptiBreech Care (non-randomised)	Total OptiBreech Care (randomised + non-randomised)
N = (%)	33	34	116	150
Gestation				
Mean at enrolment	35+6	35+5	37+4	37+1
Mean at birth	39+5	39+2	39+5	39+4
Gestational week at birth			n = 107 available	n = 141 available
36	1 (3.0%)	2 (5.9%)	1 (0.9%)	3 (2.1%)
37	4 (12.1%)	1 (2.9%)	8 (7.5%)	9 (6.4%)
38	3 (9.1%)	6 (17.6%)	15 (14.0%)	21 (14.9%)
39	13 (39.4%)	18 (52.9%)	33 (30.8%)	51 (36.2%)
40	5 (15.2%)	4 (11.8%)	33 (30.8%)	37 (26.2%)
41	4 (12.1%)	3 (8.8%)	12 (11.2%)	15 (10.6%)
42	3 (9.1%)	0	5 (4.7%)	5 (3.5%)
Source of referral				
midwife	12 (36.4%)	10 (29.4%)	48 (41.4%)	58 (38.7%)
obstetrician	3 (9.1%)	2 (5.9%)	25 (21.6%)	27 (18.0%)
sonographer	18 (54.5%)	22 (64.7%)	21 (18.1%)	43 (28.7%)
self (originally booked elsewhere)	0	0	19 (16.4%)	19 (12.7%)
another clinician / hospital	0	0	3 (2.6%)	3 (2.0%)
Diagnosis prior to labour				
prior to labour	33 (100%)	34 (100%)	105 (90.5%)	139 (92.7%)
in labour, after rupture of membranes	0	0	11 (9.5%)	11 (7.3%)
Previous vaginal births				
none	24 (72.7%)	24 (70.6%)	62 (53.4%)	86 (57.3%)
one or more	9 (27.3%)	10 (29.4%)	54 (46.6%)	64 (42.7%)
Parity				
0	21 (63.6%)	22 (64.7%)	60 (51.7%)	82 (54.7%)
1	8 (24.2%)	8 (23.5%)	41 (35.3%)	49 (32.7%)
2	3 (9.1%)	4 (11.8%)	12 (10.3%)	16 (10.7%)
3	1 (3.0%)	0	3 (2.6%)	3 (2.0%)
1 previous caesarean birth	2 (6.1%)	2 (5.9%)	5 (4.3%)	7 (4.7%)
Type of breech presentation				
extended / frank	21 (63.6%)	20 (58.8%)	60 (51.7%)	80 (53.3%)
any other or uncertain	12 (36.4%)	14 (41.2%)	56 (48.3%)	70 (46.7%)
Maternal demographics				
Age at booking (mean years, std dev)	32.4 (5.85)	32.3 (5.84)	33.2 (4.48)	33.0 (4.82)
BMI (mean, std dev)	23.2 (5.00)	25.5 (5.57)	24.2 (4.95)	24.5 (5.11)
Self-reported variables				
Gender				
female	33 (100%)	34 (100%)	116 (100%)	150 (100%)
male (trans)	0	0	0	0
non-binary	0	0	0	0
Ethnic group				
Scottish / English / Welsh / Northern Irish / British	16 (48.5%)	14 (41.2%)	57 (49.1%)	71 (47.3%)
Irish	0	1 (2.9%)	2 (1.7%)	3 (2.0%)
Any other white background	9 (27.3%)	12 (35.3%)	24 (20.7%)	36 (24.0%)
White and Black Caribbean	0	1 (2.9%)	1 (0.9%)	2 (1.3%)
White and Black African	1 (3.0%)	0	1 (0.9%)	1 (0.7%)
White and Asian	0	0	2 (1.7%)	2 (1.3%)
Any other mixed / multiple ethnic background	0	0	3 (2.6%)	3 (2.0%)
Indian	2 (6.1%)	1 (2.9%)	7 (6.0%)	8 (5.3%)
Pakistani	1 (3.0%)	0	8 (6.9%)	8 (5.3%)
Bangladeshi	1 (3.0%)	1 (2.9%)	0	1 (0.7%)
Any other Asian background	1 (3.0%)	3 (8.8%)	3 (2.6%)	6 (4.0%)
African	0	1 (2.9%)	6 (5.2%)	7 (4.7%)
Arab	2 (6.1%)	0	1 (0.9%)	1 (0.7%)
Any other ethnic group			1 (0.9%)	1 (0.7%)
Non-white-British	17 (51.5%)	20 (58.8%)	59 (50.9%)	79 (52.7%)
Black or Brown women	8 (24.2%)	7 (20.6%)	33 (28.4%)	40 (26.7%)
Interpreter required	1 (3.0%)	4 (11.8%)	3 (2.6%)	7 (4.7%)

**Table:** 

Table 1 Legend: Terms, Abbreviations and How/When data was collected	
*Gestation*	Length of pregnancy in weeks. Calculated automatically within e-CRF based on estimated date of birth provided at enrolment (via ultrasound scan or, when not available, last menstrual dates) and baby’s actual date of birth.
*Mean*	Average
*Std Dev*	Standard deviation. A quantity expressing how much the members of a group differ from the mean value for the group.
*Source of referral*	How the woman was referred for breech care and/or OptiBreech care
*Diagnosis*	When the baby is determined to be in a breech position, usually by ultrasound scan, except in labour if there is no time for an ultrasound scan to be performed.
*Extended / frank*	A breech position where both hips are flexed/bent, the baby’s legs are folded along the body with knees straight. Based on diagnosis by ultrasound scan at the time of enrolment.
*Any other position*	These include all positions where the baby’s hips or knees are flexed/bent and/or below the baby’s bottom. Based on diagnosis by ultrasound scan at the time of enrolment.
*Gender*	The person’s perceived gender-identity, which could be the same as or different from their sex. Self-reported at the time of enrolment.
*Ethnicity*	Belonging to a population or subgroup made up of people who share a common cultural background or descent. Self-reported at the time of enrolment based on the categories in the table.
*Non-white-British*	This group includes all women who indicated their ethnicity was something other than Scottish, English, Welsh, Northern Irish, British.
*Black or Brown women*	This group includes all women who indicated their ethnicity was something other than Scottish, English, Welsh, Northern Irish, British, Irish, or any other white background; and those who indicated their ethnicity was mixed.

**Table 2 Key Questions:**

1) Service leaders: How does the model of care influence the vaginal birth rate?
2) Service users: How likely am I to achieve the vaginal birth I want within OptiBreech care?

**Table 2.** Presentation on admission and mode of birth outcomes, by randomisation arm / cohort.

Outcome	Randomised Standard Care (nested RCT)	Randomised OptiBreech Care (nested RCT)	Cohort OptiBreech Care (non-randomised)	Total OptiBreech Care (randomised + non-randomised)
N (%) – *denominator listed when data* * are missing*	33	34	107	141
Presentation on admission for labour/birth				
breech	15 (45.5%)	20 (58.8%)	74/93 (79.6%)	94/127 (74.0%)
cephalic	18 (54.5%)	13 (38.2%)	19/93 (20.4%)	32/127 (25.2%)
transverse	0	1 (2.9%)	0	1/127 (0.8%)
Mode of Birth				
vaginal breech birth	0	1 (2.9%)	38 (35.5%)	39 (27.7%)
forceps breech birth	0	0	3 (2.8%)	3 (2.1%)
cephalic vaginal birth	8 (24.2%)	7 (20.6%)	12 (11.2%)	19 (13.5%)
cephalic ventouse birth	0	3 (8.8%)	1 (0.9%)	4 (2.8%)
cephalic forceps birth	0	0	0	0
in-labour caesarean birth (Cat 1/2)	12 (36.4%)	7 (20.6%)	32 (29.9%)	39 (27.7%)
pre-labour caesarean birth (Cat 3/4)	13 (39.4%)	16 (47.1%)	21 (19.6%)	37 (26.2%)
TOTAL vaginal birth	8 (24.2%)	11 (32.4%)	54 (50.5%)	65 (46.1%)
TOTAL caesarean birth	25 (75.8%)	23 (67.6%)	53 (49.5%)	76 (53.9%)
caesarean birth at full dilation	0	1/34 (2.9%)	7 (6.5%)	8 (5.7%)

**Table:** 

Table 2 Legend: Terms, Abbreviations and How/When data was collected	
*Cat 1/2*	Category 1 caesarean birth: Immediate threat to the life of woman or fetus. Category 2 caesarean birth: Maternal or fetal compromise, but not immediately life threatening. Taken from hospital records made by the care team.
*Cat 3/4*	Category 3 caesarean birth: Needing early delivery, but no maternal or fetal compromise. Category 4 caesarean birth: At a time to suit the woman and the maternity team. Taken from hospital records made by the care team.

**Table 3 Key Questions:**

1) Service leaders: How does the model of care influence neonatal outcomes?
2) Service users: How likely am I to be separated from my baby following birth? How likely is my baby to be unwell?

**Table 3.** Neonatal outcomes, by randomisation arm / cohort.

Outcome	Randomised Standard Care (nested RCT)	Randomised OptiBreech Care (nested RCT)	Cohort OptiBreech Care (non-randomised)	Total OptiBreech Care (randomised + non- randomised)
N (%) – *denominator listed when data are* * missing*	33	34	107	141
NEONATAL				
Admission to higher-level care				
Transitional care	2 (6.1%)	4 (11.8%)	13 (11.2%)	17 (12%)
NICU or SCBU prior to discharge	2 (6.1%)	2 (5.9%)	3 (2.8%)	5 (3.5%)
Adverse neonatal outcomes				
Apgar <7 at 5 minutes	0	0	2/103 (1.9%)	2/137 (1.5%)
**Severe neonatal morbidity /** ** mortality prior to discharge**	2 (6.1%)	1 (2.9%)	0	1 (0.7%)
Admission to SCBU/NICU > 4 days	1 (3.0%)	1 (2.9%)	0	2 (1.5%)
Apgar <4 at 5 minutes	0	0	0	0
HIE Grade 3	0	0	0	0
Intubation / ventilation > 24 hours	1 (3.0%)	0	0	0
Parenteral or tube feeding > 24 hours	1 (3.0%)	0	0	0
Seizures or convulsions > 24 hours	0	0	0	0
Peripheral nerve / brachial plexus injury present at discharge	0	0	0	0
Skull fracture	0	0	0	0
Spinal cord injury	0	0	0	0

**Table:** 

Table 3 Legend: Terms, Abbreviations and How/When data was collected	
*Denominator*	This is the total number of cases where we have data available. The percentage rate is calculated by dividing the number of outcomes by the number of cases available (denominator).
*Transitional care*	Neonatal transitional care supports hospital-resident mothers as primary care providers for their babies with care requirements in excess of normal newborn care, but who do not require to be in a neonatal unit. In this report, admission is measured at the time of discharge from birth care.
*NICU*	Neonatal intensive care unit. This is for babies who need the highest level of medical and nursing support. In this report, admission is measured at the time of discharge from birth care.
*SCBU*	Special care baby unit. This is a neonatal unit for babies who do not need intensive care. In this report, admission is measured at the time of discharge from birth care.
*Apgar*	A measure of the physical condition of a newborn infant. It is obtained by adding points (2, 1 or 0) for heart rate, respiratory effort, muscle tone, response to stimulation, and skin coloration. It is measured on a scale of 0-10, where 10 represents the best possible condition. It is measured at 1, 5 and 10 minutes after birth.
*Morbidity*	The state of being unwell or having a medical condition.
*Severe morbidity*	A state of being severely unwell. In this study, ‘severe morbidity’ is when one or more of the conditions listed in the above table are or have been present after birth. This is known as a ‘composite measure,’ and it is measured at the time of discharge from birth care.
*Mortality*	The state of having died. In this report, this is measured at the time the mother is discharged from birth care.
*HIE*	Hypoxic ischemic encephalopathy, a form of brain injury due to lack of oxygen before or during birth. HIE is graded on a scale ranging from 1-3, for mild, moderate, and severe cases. This study includes HIE Grade 3 (severe) as a measure of severe morbidity.
*Intubation*	Intubation is the process of inserting a tube called an endotracheal tube (ET) into the mouth or into the airway (trachea) to hold it open. This is done to assist with breathing when the baby is unable to do this on their own.
*Parenteral or tube* *feeding*	This is a way of feeding babies that cannot feed on their own. Parenteral feeding is directly into a vein. Tube feeding is done through an enteral tube, that is usually inserted through the nose and into the stomach (nasogastric tube).
*Brachial plexus injury*	The brachial plexus is a collection of nerves located between the neck and shoulders, chest, arms, hands and feeling in the upper limbs. This collection of nerves can be injured during a difficult delivery, leading to partial or complete paralysis of an arm.

**Table 4 Key Questions:**

1) Service leaders: How does the model of care influence maternal outcomes?
2) Service users: How likely am I to be unwell after my birth? What are the outcomes for my perineum likely to be?

**Table 4.** Maternal outcomes, by randomisation arm / cohort.

Outcome	Randomised Standard Care (nested RCT)	Randomised OptiBreech Care (nested RCT)	Cohort OptiBreech Care (non-randomised)	Total OptiBreech Care (randomised + non-randomised)
N (%) – *denominator listed when data are missing*	33	34	107	141
MATERNAL				
Admission to higher-level care				
HDU/ICU prior to discharge	2 (6.1%)	2 (5.9%)	4/99 (4.0%)	6/133 (4.5%)
Severe morbidity / mortality prior to discharge	5 (15.6%)	4 (11.8%)	7 (7.1%)	11 (8.3%)
Postpartum haemorrhage > 1500 mL	2 (6.0%)	1 (2.9%)	1 /99 (1.0%)	2/133 (1.5%)
Obstetric anal sphincter injury	0	0	2/98 (2.0%)	2/132 (1.5%)
Cervical laceration involving lower uterine segment	0	0	0	0
Vertical uterine incision or serious extension to transverse uterine incision	1 (3.0%)	1 (2.9%)	1/101 (1.0%)	2/135 (1.5%)
Bladder, ureter or bowel injury requiring repair	0	0	0	0
Dilation and curettage for bleeding or retained placental tissue	0	0	0	0
Manual removal of placenta	1 (3.0%)	0	2/101 (2.0%)	2/135 (1.5%)
Uterine rupture	0	0	0	0
Hysterectomy	0	0	0	0
Vulval or perineal haematoma requiring evacuation	0	0	1/101 (1.0%)	1/135 (0.7%)
Wound dehiscence / breakdown	0	0	0	0
Wound infection requiring prolonged hospital stay / readmission / antibiotics	0	1 (2.9%)	0	1 (0.7%)
Sepsis	1 (3.0%)	1 (2.9%)	0	1 (0.7%)
Disseminated intravascular coagulation	0	0	0	0
Perineal outcomes				
Intact or 1 ^st^ degree tear	29 (87.9%)	25 (73.5%)	65/98 (66.3%)	90/132 (68.2%)
2 ^nd^ degree laceration	4 (12.1%)	9 (26.5%)	31/98 (31.6%)	40/132 (30.3%)
OASI (3 ^rd^ or 4 ^th^ degree)	0	0	2/98 (2.0%)	2/132 (1.5%)
Episiotomy	0	3 (8.8%)	15/98 (15.3%)	18/132 (13.6%)

**Table:** 

Table 4 Legend: Terms, Abbreviations and How/When data was collected	
*Denominator*	This is the total number of cases where we have data available. The percentage rate is calculated by dividing the number of outcomes by the number of cases available (denominator).
*HDU*	Maternity high dependency unit. This area provides care for women needing more frequent observations than on a normal ward. It is equipped with specialist monitoring and facilities.
*ICU*	General intensive care unit. This is a specialist hospital ward that provides treatment and monitoring for people who are very ill. They are staffed with specially trained healthcare professionals and contain sophisticated monitoring equipment.
*Morbidity*	The state of being unwell or having a medical condition.
*Severe morbidity*	A state of being severely unwell. In this study, ‘severe morbidity’ is when one or more of the conditions listed in the above table are or have been present after birth. This is known as a ‘composite measure,’ and it is measured at the time of discharge following birth.
*Mortality*	The state of having died. In this report, this is measured at the time the mother is discharged following birth.
*Postpartum haemorrhage*	Excessive bleeding after birth.
*OASI*	Obstetric anal sphincter injury. A traumatic injury to the anal sphincter muscle, caused by a tear or cut during birth
*Cervical laceration*	Injury to the cervix, the opening at the bottom of the uterus (womb). This is caused by tearing or cutting.
*Vertical or extended incision*	At most caesarean births, a small side-to-side (transverse) incision (cut made during surgery) is done just above the pubic bone. If a larger incision is required, this may be top-to-bottom (vertical), or the transverse incision may extend.
*Uterine rupture*	A tear in the muscular wall of the uterus that occurs during pregnancy or childbirth, in which the baby slips out into the abdomen.
*Hysterectomy*	Removal of the uterus.
*Haematoma*	A collection of blood outside the major blood vessels.
*Wound dehiscence*	This is a complication of surgery or perineal suturing where the laceration or incision reopens. It is a partial separation of the wound edges due to lack of proper wound healing.
*Sepsis*	Sepsis is a serious condition that happens when the body’s immune system has an extreme response to an infection. The body’s reaction causes damage to its own tissues and organs.
*Disseminated intravascular* *coagulation*	DIC. This is a serious disorder occurring in response to excessive bleeding or other disease process that results in blood clotting not functioning normally.

**Analysis by first plan following counselling (entire cohort)**

**Table 5 Key Questions:**

1) Service leaders: How does an ECV service affect the vaginal birth rate?
2) Service users: How likely am I to have the type of birth I choose to plan?

**Table 5.** Presentation on admission and mode of birth outcomes, by first plan following counselling.

Outcome	Plan following first counselling (entire cohort)				
	Vaginal breech birth	Attempt at ECV	Pre-labour caesarean birth	CB in early labour (<3 cm)	CB in active labour (>3 cm)*
N (%) – *denominator listed * *when data are missing*	42	115	14	1	2
Presentation on admission for labour/birth					
breech	35/38 (92.1%)	59/105 (56.2%)	13 (92.9%)	1 (100%)	1 (50%)
cephalic	3/38 (7.9%)	45/105 (42.9%)	1 (7.1%)	0	1 (50%)*
transverse	0	1/105 (1.0%)	0	0	0
Mode of Birth					
vaginal breech birth	16 (38.1%)	19 (16.5%)	1 (7.1%)	1 (100%)	2 (100%)
forceps breech birth	1 (2.4%)	2 (1.7%)	0	0	0
cephalic vaginal birth	3 (7.1%)	24 (20.9%)	0	0	0
cephalic ventouse birth	0	4 (3.5%)	0	0	0
cephalic forceps birth	0	0	0	0	0
in-labour caesarean birth	15 (35.7%)	36 (31.3%)	0	0	0
pre-labour caesarean birth	7 (16.7%)	30 (26.1%)	13 (92.9%)	0	0
TOTAL vaginal birth	20 (47.6%)	49 (42.6%)	1 (7.1%)	1 (100%)	2 (100%)
TOTAL caesarean birth	22 (52.4%)	66 (57.4%)	13 (92.9%)	0	0
caesarean birth at full dilation	4/42 (9.5%)	4/115 (3.5%)	0	0	0

**Table:** 

Table 5 Legend: Terms, Abbreviations and How/When data was collected	
*ECV*	External cephalic version. An attempt to turn the baby to a head-down position in the womb, using manual pressure on the maternal abdomen.
*CB*	Caesarean birth.
*Cat 1/2*	Category 1 caesarean birth: Immediate threat to the life of woman or fetus. Category 2 caesarean birth: Maternal or fetal compromise, but not immediately life threatening. Taken from hospital records made by the care team.
*Cat 3/4*	Category 3 caesarean birth: Needing early delivery, but no maternal or fetal compromise. Category 4 caesarean birth: At a time to suit the woman and the maternity team. Taken from hospital records made by the care team.
*	Undiagnosed breech. Breech presentation diagnosed for the first time in labour; documented as cephalic on admission.

**Table 6 Key Questions:**

1) Service leaders: How does an ECV service affect neonatal outcomes?
2) Service users: How will my care choices influence outcomes for my baby?

**Table 6.** Neonatal outcomes, by first plan following counselling (entire cohort).

Outcome	Plan following first counselling (entire cohort)				
	Vaginal breech birth	Attempt at ECV	Pre-labour caesarean birth	CB in early labour (<3 cm)	CB in active labour (>3 cm)*
N (%) – *denominator listed when data are* * missing*	42	115	14	1	2
NEONATAL					
Admission to higher-level care					
Transitional care	3 (7.1%)	14 (12.0%)	1 (7.1%)	0	1 (50%)
NICU or SCBU prior to discharge	1 (2.4%)	5 (4.3%)	0	0	1 (50%)
Adverse outcomes					
Apgar <7 at 5 minutes	2/40 (5.0%)	0	0	0	0
Severe morbidity / mortality prior to discharge	0	3 (2.6%)	0	0	0
Admission to SCBU/NICU > 4 days	0	2 (1.7%)	0	0	0
Apgar <4 at 5 minutes	0	0	0	0	0
HIE Grade 3	0	0	0	0	0
Intubation / ventilation > 24 hours	0	1 (0.9%)	0	0	0
Parenteral or tube feeding > 24 hours	0	1 (0.9%)	0	0	0
Seizures or convulsions > 24 hours	0	0	0	0	0
Peripheral nerve / brachial plexus injury present at discharge	0	0	0	0	0
Skull fracture	0	0	0	0	0
Spinal cord injury	0	0	0	0	0

**Table:** 

Table 6 Legend: Terms, Abbreviations and How/When data was collected	
*ECV*	External cephalic version. An attempt to turn the baby to a head-down position in the womb, using manual pressure on the maternal abdomen.
*CB*	Caesarean birth.
*Denominator*	This is the total number of cases where we have data available. The percentage rate is calculated by dividing the number of outcomes by the number of cases available (denominator).
*	Undiagnosed breech. Breech presentation diagnosed for the first time in labour; documented as cephalic on admission.
*Transitional care*	Neonatal transitional care supports hospital-resident mothers as primary care providers for their babies with care requirements in excess of normal newborn care, but who do not require to be in a neonatal unit. In this report, admission is measured at the time of discharge from birth care.
*NICU*	Neonatal intensive care unit. This is for babies who need the highest level of medical and nursing support. In this report, admission is measured at the time of discharge from birth care.
*SCBU*	Special care baby unit. This is a neonatal unit for babies who do not need intensive care. In this report, admission is measured at the time of discharge from birth care.
*Apgar*	A measure of the physical condition of a newborn infant. It is obtained by adding points (2, 1 or 0) for heart rate, respiratory effort, muscle tone, response to stimulation, and skin coloration. It is measured on a scale of 0-10, where 10 represents the best possible condition. It is measured at 1, 5 and 10 minutes after birth.
*Morbidity*	The state of being unwell or having a medical condition.
*Severe morbidity*	A state of being severely unwell. In this study, ‘severe morbidity’ is when one or more of the conditions listed in the above table are or have been present after birth. This is known as a ‘composite measure,’ and it is measured at the time of discharge from birth care.
*Mortality*	The state of having died. In this report, this is measured at the time the mother is discharged from birth care.
*HIE*	Hypoxic ischemic encephalopathy. HIE is graded on a scale ranging from 1-3, for mild, moderate and severe cases. This study includes HIE Grade 3 (severe) as a measure of severe morbidity.
*Intubation*	Intubation is the process of inserting a tube called an endotracheal tube (ET) into the mouth or into the airway (trachea) to hold it open. This is done to assist with breathing when the baby is unable to do this on their own.
*Parenteral or tube* *feeding*	This is a way of feeding babies that cannot feed on their own. Parenteral feeding is directly into a vein. Tube feeding is done through an enteral tube, that is usually inserted through the nose and into the stomach (nasogastric tube).
*Brachial plexus injury*	The brachial plexus is a collection of nerves located between the neck and shoulders, chest, arms, hands and feeling in the upper limbs. This collection of nerves can be injured during a difficult delivery, leading to partial or complete paralysis of an arm.

**Table 7 Key Questions:**

1) Service leaders: How does an ECV service affect maternal outcomes?
2) Service users: How will my care choices influence my health after birth?

**Table 7.** Maternal outcomes, by first plan following counselling (entire cohort).

Outcome	Plan following first counselling (entire cohort)				
	Vaginal breech birth	Attempt at ECV	Pre-labour caesarean birth	CB in early labour (<3 cm)	CB in active labour (>3 cm)*
N (%) – *denominator listed when data are missing*	42	115	14	1	2
MATERNAL					
Admission to higher-level care					
HDU	2/37 (5.4%)	5/111 (4.5%)	0	0	1 (50%)
Severe morbidity / mortality prior to discharge	2/37 (5.4%)	12/111 (10.8%)	2 (14.3%)	0	0
Postpartum haemorrhage > 1500 mL	0	3/111 (2.7%)	1 (7.1%)	0	0
Obstetric anal sphincter injury	1/38 (2.6%)	1/111 (0.9%)	0	0	0
Cervical laceration involving lower uterine segment	0	0	0	0	0
Vertical uterine incision or serious extension to transverse uterine incision	0	3/112 (2.7%)	0	0	0
Bladder, ureter or bowel injury requiring repair	0	0	0	0	0
Dilation and curettage for bleeding or retained placental tissue	0	0	0	0	0
Manual removal of placenta	1/39 (2.6%)	2/112 (1.8%)	0	0	0
Uterine rupture	0	0	0	00	
Hysterectomy	0	0	0	0	0
Vulval or perineal haematoma requiring evacuation	0	1/112 (0.9%)	0	0	0
Wound dehiscence / breakdown	0	0	0	0	0
Wound infection requiring prolonged hospital stay / readmission / antibiotics	0	0	1 (7.1%)	0	0
Sepsis	0	2/112 (1.8%)	0	0	0
Disseminated intravascular coagulation	0	0	0	0	0
Perineal outcomes					
Intact or 1 ^st^ degree tear	24/38 (63.2%)	81/111 (73.0%)	13/13 (100%)	1/1 (100%)	0
2 ^nd^ degree laceration	13/38 (34.2%)	29/112 (25.9%)	0	0	2/2 (100%)
OASI (3 ^rd^ or 4 ^th^ degree tear)	1/38 (2.6%)	1/112 (0.9%)	0	0	0
Episiotomy	7/38 (18.4%)	9/112 (8.0%)	0	0	2/2 (100%)

**Table:** 

Table 7 Legend: Terms, Abbreviations and How/When data was collected	
*ECV*	External cephalic version. An attempt to turn the baby to a head-down position in the womb, using manual pressure on the maternal abdomen.
*CB*	Caesarean birth.
*Denominator*	This is the total number of cases where we have data available. The percentage rate is calculated by dividing the number of outcomes by the number of cases available (denominator).
*	Undiagnosed breech. Breech presentation diagnosed for the first time in labour; documented as cephalic on admission.
*HDU*	Maternity high dependency unit. This area provides care for women needing more frequent observations than on a normal ward. It is equipped with specialist monitoring and facilities.
*ICU*	General intensive care unit. This is a specialist hospital ward that provides treatment and monitoring for people who are very ill. They are staffed with specially trained healthcare professionals and contain sophisticated monitoring equipment.
*Morbidity*	The state of being unwell or having a medical condition.
*Severe morbidity*	A state of being severely unwell. In this study, ‘severe morbidity’ is when one or more of the conditions listed in the above table are or have been present after birth. This is known as a ‘composite measure,’ and it is measured at the time of discharge following birth.
*Mortality*	The state of having died. In this report, this is measured at the time the mother is discharged following birth.
*Postpartum haemorrhage*	Excessive bleeding after birth.
*OASI*	Obstetric anal sphincter injury. A traumatic injury to the anal sphincter muscle, caused by a tear or cut during birth
*Cervical laceration*	Injury to the cervix, the opening at the bottom of the uterus (womb). This is caused by tearing or cutting.
*Vertical or extended incision*	At most caesarean births, a small side-to-side (transverse) incision (cut made during surgery) is done just above the pubic bone. If a larger incision is required, this may be top-to-bottom (vertical), or the transverse incision may extend.
*Uterine rupture*	A tear in the muscular wall of the uterus that occurs during pregnancy or childbirth, in which the baby slips out into the abdomen.
*Hysterectomy*	Removal of the uterus.
*Haematoma*	A collection of blood outside the major blood vessels.
*Wound dehiscence*	This is a complication of surgery or perineal suturing where the laceration or incision reopens. It is a partial separation of the wound edges due to lack of proper wound healing.
*Sepsis*	Sepsis is a serious condition that happens when the body’s immune system has an extreme response to an infection. The body’s reaction causes damage to its own tissues and organs.
*Disseminated intravascular coagulation*	DIC. This is a serious disorder occurring in response to excessive bleeding or other disease process that results in blood clotting not functioning normally.

### Analysis by planned mode of birth / actual mode of birth / presentation on admission

**Table 8 Key Questions:**

1) Service leaders: How does achieving cephalic presentation at birth affect vaginal birth rates?
2) Service users: How likely am I to have the type of birth I choose to plan? How will my baby’s position at the time of birth influence this?

**Table 8.** Mode of birth outcomes, by planned birth / presentation on admission.

Outcome	Planned vaginal breech birth at any point (all OptiBreech)	Did not plan a vaginal breech birth at any point (entire cohort)	Planned cephalic birth (entire cohort)	Actual vaginal births		Presentation on admission for labour/birth (entire cohort)		
				Breech (all OptiBreech)	Cephalic (entire cohort)	breech	cephalic	transverse
N (%) – *denominator listed * *when data are missing*	97	77	54	42	31	109	50	1
Mode of Birth								
vaginal breech birth	37/97 (38.1%)	2/77 (2.6%)	3/54 (5.6%)	39/42 (92.9%)		34/109 (31.2%)	3/50 (6.0%) *	0
forceps breech birth	3/97 (3.1%)	0	0	3/42 (7.1%)		3/109 (2.8%)	0	0
cephalic vaginal birth	4/97 (4.1%)	23/77 (29.9%)	25/54 (46.3%)		27/31 (87.1%)	0	24/50 (48.0%)	1
cephalic ventouse birth	0	4/77 (5.2%)	4/54 (7.4%)		4/31 (12.9%)	0	3/50 (6.0%)	0
cephalic forceps birth	0	0	0		0	0	0	0
in-labour caesarean birth	27/97 (27.8%)	24/77 (31.2%)	20/54 (37.0%)			29/109 (26.6%)	18/50 (36.0%)	0
pre-labour caesarean birth	26/97 (26.8%)	24/77 (31.2%)	2/54 (3.7%)			43/109 (39.4%)	2/50 (4.0%)	0
TOTAL vaginal birth	44/97 (45.4%)	29/77 (37.7%)	32/54 (59.3%)			37/109 (33.9%)	30/50 (60.0%)	1
TOTAL caesarean birth	53/97 (54.6%)	48/77 (62.3%)	22/54 (40.7%)			72/109 (66.1%)	20/50 (40.0%)	0
caesarean birth at full dilation	6/97 (6.2%)	2/77 (2.6%)	2/54 (3.7%)			6/109 (5.5%)	2/50 (4.0%)	0

**Table:** 

Table 8 Legend: Terms, Abbreviations and How/When data was collected	
*CB*	Caesarean birth.
*Cat 1/2*	Category 1 caesarean birth: Immediate threat to the life of woman or fetus. Category 2 caesarean birth: Maternal or fetal compromise, but not immediately life threatening. Taken from hospital records made by the care team.
*Cat 3/4*	Category 3 caesarean birth: Needing early delivery, but no maternal or fetal compromise. Category 4 caesarean birth: At a time to suit the woman and the maternity team. Taken from hospital records made by the care team.
*	Undiagnosed breech. Breech presentation diagnosed for the first time in labour; documented as cephalic on admission.

**Table 9 Key Questions:**

1) Service leaders: How does achieving cephalic presentation at birth affect neonatal outcomes?
2) Service users: How will my care choices influence outcomes for my baby?

**Table 9.** Neonatal outcomes, by planned birth / presentation on admission.

Outcome	Planned vaginal breech birth at any point (all OptiBreech)	Did not plan a vaginal breech birth at any point (entire cohort)	Planned cephalic birth (entire cohort)	Actual vaginal births		Presentation on admission for labour/birth (entire cohort)		
				Breech (all OptiBreech)	Cephalic (entire cohort)	breech	cephalic	transverse
N (%) – *denominator listed* *when data are missing*	97	77	54	42	31	109	50	1
NEONATAL								
Admission to higher- level care								
Transitional care	10 (10.3%)	9 (11.7%)	10 (18.5%)	4 (9.5%)	5 (16.1%)	9 (8.3%)	9 (18.0%)	0
NICU or SCBU prior to discharge	4 (4.1%)	3 (3.9%)	3 (5.6%)	2 (4.8%)	2 (6.5%)	4 (3.7%)	3 (6.0%)	0
Adverse outcomes								
Apgar <7 at 5 minutes	2/93 (2.2%)	0	0	1 (2.4%)	0	2 (1.8%)	0	0
Severe morbidity / mortality prior to discharge	0	3/77 (3.9%)	2 (3.7%)	0	1 (3.2%)	1 (0.9%)	2 (4.0%)	0
Admission to SCBU/ NICU > 4 days	0	2 (2.6%)	1 (1.9%)	0	1 (3.2%)	1 (0.9%)	1 (2.0%)	0
Apgar <4 at 5 minutes	0	0	0	0	0	0	0	0
HIE Grade 3	0	0	0	0	0	0	0	0
Intubation / ventilation > 24 hours	0	1 (1.3%)	0	0	0	1 (0.9%)	0	0
Parenteral or tube feeding > 24 hours	0	1 (1.3%)	1 (1.9%)	0	0	0	1 (2.0%)	0
Seizures or convulsions > 24 hours	0	0	0	0	0	0	0	0
Peripheral nerve / brachial plexus injury present at discharge	0	0	0	0	0	0	0	0
Skull fracture	0	0	0	0	0	0	0	0
Spinal cord injury	0	0	0	0	0	0	0	0

**Table:** 

Table 9 Legend: Terms, Abbreviations and How/When data was collected	
*Denominator*	This is the total number of cases where we have data available. The percentage rate is calculated by dividing the number of outcomes by the number of cases available (denominator).
*Transitional care*	Neonatal transitional care supports hospital-resident mothers as primary care providers for their babies with care requirements in excess of normal newborn care, but who do not require to be in a neonatal unit. In this report, admission is measured at the time of discharge from birth care.
*NICU*	Neonatal intensive care unit. This is for babies who need the highest level of medical and nursing support. In this report, admission is measured at the time of discharge from birth care.
*SCBU*	Special care baby unit. This is a neonatal unit for babies who do not need intensive care. In this report, admission is measured at the time of discharge from birth care.
*Apgar*	A measure of the physical condition of a newborn infant. It is obtained by adding points (2, 1 or 0) for heart rate, respiratory effort, muscle tone, response to stimulation, and skin coloration. It is measured on a scale of 0-10, where 10 represents the best possible condition. It is measured at 1, 5 and 10 minutes after birth.
*Morbidity*	The state of being unwell or having a medical condition.
*Severe morbidity*	A state of being severely unwell. In this study, ‘severe morbidity’ is when one or more of the conditions listed in the above table are or have been present after birth. This is known as a ‘composite measure,’ and it is measured at the time of discharge from birth care.
*Mortality*	The state of having died. In this report, this is measured at the time the mother is discharged from birth care.
*HIE*	Hypoxic ischemic encephalopathy. HIE is graded on a scale ranging from 1-3, for mild, moderate and severe cases. This study includes HIE Grade 3 (severe) as a measure of severe morbidity.
*Intubation*	Intubation is the process of inserting a tube called an endotracheal tube (ET) into the mouth or into the airway (trachea) to hold it open. This is done to assist with breathing when the baby is unable to do this on their own.
*Parenteral or tube* *feeding*	This is a way of feeding babies that cannot feed on their own. Parenteral feeding is directly into a vein. Tube feeding is done through an enteral tube, that is usually inserted through the nose and into the stomach (nasogastric tube).
*Brachial plexus* *injury*	The brachial plexus is a collection of nerves located between the neck and shoulders, chest, arms, hands and feeling in the upper limbs. This collection of nerves can be injured during a difficult delivery, leading to partial or complete paralysis of an arm.

**Table 10 Key Questions:**

1) Service leaders: How does achieving cephalic presentation at birth affect maternal outcomes?
2) Service users: How will my care choices influence my health after birth?

**Table 10.** Maternal outcomes, by planned birth / presentation on admission.

Outcome	Planned vaginal breech birth at any point (all OptiBreech)	Did not plan a vaginal breech birth at any point (entire cohort)	Planned cephalic birth (entire cohort)	Actual vaginal births		Presentation on admission for labour/birth (entire cohort)		
				Breech (all OptiBreech)	Cephalic (entire cohort)	breech	cephalic	transverse
N (%) – *denominator listed * *when data are missing*	97	77	54	42	31	109	50	1
MATERNAL								
Admission to higher- level care								
HDU	2 (2.1%)	6 (7.8%)	4 (7.4%)	1 (2.4%)	1 (3.2%)	4 (3.7%)	3 (6.0%)	0
Severe morbidity / mortality prior to discharge	7/89 (7.9%)	9/76 (11.8%)	8 (14.8%)	5/41 (12.2%)	3/30 (10.0%)	8/105 (7.6%)	7 (14.0%)	0
Postpartum haemorrhage > 1500 mL	0	4/76 (5.3%)	3 (5.6%)	0	1/30 (3.3%)	1/105 (1.0%)	2 (4.0%)	0
Obstetric anal sphincter injury	2/88 (2.3%)	0	1 (1.9%)	2/39 (5.1%)	0	1/105 (1.0%)	1/50 (2.0%)*	0
Cervical laceration involving lower uterine segment	0	0	0	0	0	0	0	0
Vertical uterine incision or serious extension to transverse uterine incision	2/91 (2.2%)	1 (1.3%)	1 (1.9%)	0	0	2/108 (1.9%)	1/50 (2.0%)	0
Bladder, ureter or bowel injury requiring repair	0	0	0	0	0	0	0	0
Dilation and curettage for bleeding or retained placental tissue	0	0	0	0	0	0	0	0
Manual removal of placenta	2/91 (2.2%)	1 (1.3%)	1 (1.9%)	2/41 (4.9%)	1/30 (3.3%)	2/108 (1.9%)	1/50 (2.0%)	0
Uterine rupture	0	0	0	0	0	0	0	0
Hysterectomy	0	0	0	0	0	0	0	0
Vulval or perineal haematoma requiring evacuation	1/91 (1.1%)	0	0	1/41 (2.4%)	0	1/108 (0.9%)	0	0
Wound dehiscence / breakdown	0	0	0	0	0	0	0	0
Wound infection requiring prolonged hospital stay / readmission / antibiotics	0	1 (0.9%)	0	0	0	1/108 (0.9%)	0	0
Sepsis	0	2 (2.6%)	2 (3.7%)	0	1 (3.2%)	0	2 (4.0%)	0
Disseminated intravascular coagulation	0	0	0	0	0	0	0	0
Perineal outcomes								
Intact or 1 ^st^ degree tear	62/88 (70.5%)	57 (74.0%)	31 (57.4%)	14/39 (35.9%)	9/30 (30.0%)	83/105 (79.0%)	29/50 (58.0%)	0
2 ^nd^ degree tear	24/88 (27.2%)	20 (26.0%)	22 (40.7%)	23/39 (59.0%)	21/30 (70%)	21/105 (20.0%)	20/50 (40.0%)	1/1 (100%)
OASI (3 ^rd^ or 4 ^th^ degree tear)	2/88 (2.3%)	0	1 (1.9%)	2/39 (4.8%)	0	1/105 (1.0%)	1/50 (2.0%)*	0
Episiotomy	13/88 (14.8%)	5 (6.5%)	6 (11.1%)	14 (33.3%)	4 (12.9%)	12/105 (11.4%)	5/50 (10.0%)	0

**Table:** 

Table 10 Legend: Terms, Abbreviations and How/When data was collected	
*Denominator*	This is the total number of cases where we have data available. The percentage rate is calculated by dividing the number of outcomes by the number of cases available (denominator).
*HDU*	Maternity high dependency unit. This area provides care for women needing more frequent observations than on a normal ward. It is equipped with specialist monitoring and facilities.
*ICU*	General intensive care unit. This is a specialist hospital ward that provides treatment and monitoring for people who are very ill. They are staffed with specially trained healthcare professionals and contain sophisticated monitoring equipment.
*Morbidity*	The state of being unwell or having a medical condition.
*Severe morbidity*	A state of being severely unwell. In this study, ‘severe morbidity’ is when one or more of the conditions listed in the above table are or have been present after birth. This is known as a ‘composite measure,’ and it is measured at the time of discharge following birth.
*Mortality*	The state of having died. In this report, this is measured at the time the mother is discharged following birth.
*Postpartum* * haemorrhage*	Excessive bleeding after birth.
*OASI*	Obstetric anal sphincter injury. A traumatic injury to the anal sphincter muscle, caused by a tear or cut during birth
*Cervical laceration*	Injury to the cervix, the opening at the bottom of the uterus (womb). This is caused by tearing or cutting.
*Vertical or extended* * incision*	At most caesarean births, a small side-to-side (transverse) incision (cut made during surgery) is done just above the pubic bone. If a larger incision is required, this may be top-to-bottom (vertical), or the transverse incision may extend.
*Uterine rupture*	A tear in the muscular wall of the uterus that occurs during pregnancy or childbirth, in which the baby slips out into the abdomen.
*Hysterectomy*	Removal of the uterus.
*Haematoma*	A collection of blood outside the major blood vessels.
*Wound dehiscence*	This is a complication of surgery or perineal suturing where the laceration or incision reopens. It is a partial separation of the wound edges due to lack of proper wound healing.
*Sepsis*	Sepsis is a serious condition that happens when the body’s immune system has an extreme response to an infection. The body’s reaction causes damage to its own tissues and organs.
*Disseminated* * intravascular* * coagulation*	DIC. This is a serious disorder occurring in response to excessive bleeding or other disease process that results in blood clotting not functioning normally.

### Analysis by ethnicity

**Table 11 Key Questions:**

1) Service leaders: How do vaginal birth rates compare between ethnic groups in this cohort?
2) Service users: How do vaginal birth rates compare between ethnic groups?

**Table 11.** Mode of birth outcomes, by ethnicity.

Outcome	White British participants	Non-White British ethnicity	White participants	Black or Brown participants
N (%) – *denominator listed when data* * are missing*	87/183 (47.5%)	96/183 (52.5%)	135/183 (73.8%)	48/183 (26.2%)
Presentation on admission for labour/birth				
breech	54/76 (71.1%)	55/84 (65.5%)	82/116 (70.7%)	27/44 (61.4%)
cephalic	22/76 (28.9%)	28/84 (33.3%)	34/116 (29.3%)	16/44 (36.4%)
transverse	0	1/84 (1.2%)	0	1/44 (2.3%)
vaginal breech birth	19/83 (22.9%)	20/91 (22.0%)	23/128 (18.0%)	16/46 (34.8%)
forceps breech birth	3/83 (3.6%)	0	3/128 (2.3%)	0
cephalic vaginal birth	13/83 (15.7%)	14/91 (15.4%)	21/128 (16.4%)	6/46 (13.0%)
cephalic ventouse birth	3/83 (3.6%)	1/91 (1.1%)	4/128 (3.1%)	0
cephalic forceps birth	0	0	0	0
in-labour caesarean birth (Cat 1/2)	26/83 (31.3%)	25/91 (27.5%)	37/128 (28.9%)	14/46 (30.4%)
pre-labour caesarean birth (Cat 3/4)	19/83 (22.9%)	31/91 (34.1%)	40/128 (31.3%)	10/46 (21.7%)
TOTAL vaginal birth	38/83 (45.8%)	35/91 (38.5%)	51 /128 (39.8%)	22/46 (47.8%)
TOTAL caesarean birth	45/83 (54.2%)	56/91 (61.5%)	77/128 (60.2%)	24/46 (52.2%)
caesarean birth at full dilation	7/75 (9.3%)	1/82 (1.2%)	7/115 (6.1%)	1/82 (1.2%)

**Table:** 

Table 11 Legend: Terms, Abbreviations and How/When data was collected	
*Cat 1/2*	Category 1 caesarean birth: Immediate threat to the life of woman or fetus. Category 2 caesarean birth: Maternal or fetal compromise, but not immediately life threatening. Taken from hospital records made by the care team.
*Cat 3/4*	Category 3 caesarean birth: Needing early delivery, but no maternal or fetal compromise. Category 4 caesarean birth: At a time to suit the woman and the maternity team. Taken from hospital records made by the care team.

**Table 12 Key Questions:**

1) Service leaders: How does ethnicity influence neonatal outcomes within an OptiBreech care model?
2) Service users: How will my ethnicity affect outcomes for my baby within an OptiBreech care model?

**Table 12.** Neonatal outcomes, by ethnicity.

Outcome	White British participants	Non-White British ethnicity	White participants	Black or Brown participants
N (%) – *denominator listed when data are missing*	87/183 (47.5%)	96/183 (52.5%)	135/183 (73.8%)	48/183 (26.2%)
NEONATAL				
Admission to higher-level care				
Transitional care	6/87 (6.9%)	13/96 (13.5%)	13/135 (9.6%)	6/48 (12.5%)
NICU or SCBU prior to discharge	5/83 (6.0%)	2/91 (2.2%)	5/128 (3.9%)	2/46 (4.3%)
Adverse neonatal outcomes				
Apgar <7 at 5 minutes	1/81 (1.2%)	1/89 (1.1%)	2/125 (1.6%)	0
**Severe neonatal morbidity / mortality prior to discharge**	3/82 (3.7%)	0	3/126 (2.4%)	0
Admission to SCBU/NICU > 4 days	2/83 (2.4%)	0	2/128 (1.6%)	0
Apgar <4 at 5 minutes	0	0	0	0
HIE Grade 3	0	0	0	0
Intubation / ventilation > 24 hours	1/83 (1.2%)	0	1/128 (0.8%)	0
Parenteral or tube feeding > 24 hours	1/83 (1.2%)	0	1/128 (0.8%)	0
Seizures or convulsions > 24 hours	0	0	0	0
Peripheral nerve / brachial plexus injury present at discharge	0	0	0	0
Skull fracture	0	0	0	0
Spinal cord injury	0	0	0	0

**Table:** 

Table 12 Legend: Terms, Abbreviations and How/When data was collected	
*Denominator*	This is the total number of cases where we have data available. The percentage rate is calculated by dividing the number of outcomes by the number of cases available (denominator).
*Transitional care*	Neonatal transitional care supports hospital-resident mothers as primary care providers for their babies with care requirements in excess of normal newborn care, but who do not require to be in a neonatal unit. In this report, admission is measured at the time of discharge from birth care.
*NICU*	Neonatal intensive care unit. This is for babies who need the highest level of medical and nursing support. In this report, admission is measured at the time of discharge from birth care.
*SCBU*	Special care baby unit. This is a neonatal unit for babies who do not need intensive care. In this report, admission is measured at the time of discharge from birth care.
*Apgar*	A measure of the physical condition of a newborn infant. It is obtained by adding points (2, 1 or 0) for heart rate, respiratory effort, muscle tone, response to stimulation, and skin coloration. It is measured on a scale of 0-10, where 10 represents the best possible condition. It is measured at 1, 5 and 10 minutes after birth.
*Morbidity*	The state of being unwell or having a medical condition.
*Severe morbidity*	A state of being severely unwell. In this study, ‘severe morbidity’ is when one or more of the conditions listed in the above table are or have been present after birth. This is known as a ‘composite measure,’ and it is measured at the time of discharge from birth care.
*Mortality*	The state of having died. In this report, this is measured at the time the mother is discharged from birth care.
*HIE*	Hypoxic ischemic encephalopathy. HIE is graded on a scale ranging from 1-3, for mild, moderate, and severe cases. This study includes HIE Grade 3 (severe) as a measure of severe morbidity.
*Intubation*	Intubation is the process of inserting a tube called an endotracheal tube (ET) into the mouth or into the airway (trachea) to hold it open. This is done to assist with breathing when the baby is unable to do this on their own.
*Parenteral or tube* *feeding*	This is a way of feeding babies that cannot feed on their own. Parenteral feeding is directly into a vein. Tube feeding is done through an enteral tube, that is usually inserted through the nose and into the stomach (nasogastric tube).
*Brachial plexus* *injury*	The brachial plexus is a collection of nerves located between the neck and shoulders, chest, arms, hands, and feeling in the upper limbs. This collection of nerves can be injured during a difficult delivery, leading to partial or complete paralysis of an arm.

**Table 13 Key Questions:**

1) Service leaders: How does ethnicity influence maternal outcomes within an OptiBreech care model?
2) Service users: How will my ethnicity affect my health after birth within an OptiBreech care model?

**Table 13.** Maternal outcomes, by ethnicity.

Outcome	White British participants	Non-White British ethnicity	White participants	Black or Brown participants
N (%) – *denominator listed when data are missing*	87/183 (47.5%)	96/183 (52.5%)	135/183 (73.8%)	48/183 (26.2%)
MATERNAL				
Admission to higher-level care				
HDU/ICU prior to discharge	3/87 (3.4%)	5/96 (5.2%)	4/135 (3.0%)	4/48 (8.3%)
Severe morbidity / mortality prior to discharge	6/79 (7.6%)	10/86 (11.6%)	10/122 (8.2%)	6/43 (14.0%)
Postpartum haemorrhage > 1500 mL	2/79 (2.5%)	2/86 (2.3%)	3/122 (2.5%)	1/43 (2.3%)
Obstetric anal sphincter injury	0	2/86 (2.3%)	0	2/43 (4.7%)
Cervical laceration involving lower uterine segment	0	0	0	0
Vertical uterine incision or serious extension to transverse uterine incision	1/81 (1.2%)	2/87 (2.3%)	2/124 (1.6%)	1/44 (2.3%)
Bladder, ureter or bowel injury requiring repair	0	0	0	0
Dilation and curettage for bleeding or retained placental tissue	0	0	0	0
Manual removal of placenta	1/81 (1.2%)	2/87 (2.3%)	2/124 (1.6%)	1/44 (2.3%)
Uterine rupture	0	0	0	0
Hysterectomy	0	0	0	0
Vulval or perineal haematoma requiring evacuation	0	1/87 (1.1%)	0	1/44 (2.3%)
Wound dehiscence / breakdown	0	0	0	0
Wound infection requiring prolonged hospital stay / readmission / antibiotics	1/81 (1.2%)	0	1/124 (0.8%)	0
Sepsis	1/81 (1.2%)	1/87 (1.1%)	2/124 (1.6%)	0
Disseminated intravascular coagulation	0	0	0	0
Perineal outcomes				
Intact or 1 ^st^ degree tear	56/79 (70.9%)	63/86 (73.3%)	91/122 (74.6%)	28/43 (65.1%)
2 ^nd^ degree laceration	23/79 (29.1%)	21/86 (24.4%)	31/122 (25.4%)	13/43 (30.2%)
OASI (3 ^rd^ or 4 ^th^ degree)	0	2/86 (2.3%)	0	2/43 (4.7%)
Episiotomy	12/79 (15.2%)	6/86 (7.0%)	15/122 (12.3%)	3/43 (7.0%)

**Table:** 

Table 13 Legend: Terms, Abbreviations and How/When data was collected	
*Denominator*	This is the total number of cases where we have data available. The percentage rate is calculated by dividing the number of outcomes by the number of cases available (denominator).
*HDU*	Maternity high dependency unit. This area provides care for women needing more frequent observations than on a normal ward. It is equipped with specialist monitoring and facilities.
*ICU*	General intensive care unit. This is a specialist hospital ward that provides treatment and monitoring for people who are very ill. They are staffed with specially trained healthcare professionals and contain sophisticated monitoring equipment.
*Morbidity*	The state of being unwell or having a medical condition.
*Severe morbidity*	A state of being severely unwell. In this study, ‘severe morbidity’ is when one or more of the conditions listed in the above table are or have been present after birth. This is known as a ‘composite measure,’ and it is measured at the time of discharge following birth.
*Mortality*	The state of having died. In this report, this is measured at the time the mother is discharged following birth.
*Postpartum haemorrhage*	Excessive bleeding after birth.
*OASI*	Obstetric anal sphincter injury. A traumatic injury to the anal sphincter muscle, caused by a tear or cut during birth
*Cervical laceration*	Injury to the cervix, the opening at the bottom of the uterus (womb). This is caused by tearing or cutting.
*Vertical or extended incision*	At most caesarean births, a small side-to-side (transverse) incision (cut made during surgery) is done just above the pubic bone. If a larger incision is required, this may be top-to-bottom (vertical), or the transverse incision may extend.
*Uterine rupture*	A tear in the muscular wall of the uterus that occurs during pregnancy or childbirth, in which the baby slips out into the abdomen.
*Hysterectomy*	Removal of the uterus.
*Haematoma*	A collection of blood outside the major blood vessels.
*Wound dehiscence*	This is a complication of surgery or perineal suturing where the laceration or incision reopens. It is a partial separation of the wound edges due to lack of proper wound healing.
*Sepsis*	Sepsis is a serious condition that happens when the body’s immune system has an extreme response to an infection. The body’s reaction causes damage to its own tissues and organs.
*Disseminated intravascular* *coagulation*	DIC. This is a serious disorder occurring in response to excessive bleeding or other disease process that results in blood clotting not functioning normally.

### Infant feeding outcomes

**Table 14 Key Questions:**

1) Service leaders: How does provision of OptiBreech collaborative care influence women’s ability to breastfeed when they have chosen this?
2) Service users: How will OptiBreech care influence my ability to feed my baby the way I have chosen?

**Table 14.** Infant feeding outcomes, by cohort and planned mode of birth.

	All standard care	All OptiBreech care	Planned VBB at any point	Planned cephalic birth
N (%) – *denominator listed when data are missing*	33	137	94	51
Self-reported feeding intentions				
Breast milk (or expressed breast milk) only	22 (66.7%)	111 (81.0%)	79 (84.0%)	41 (80.4%)
Formula milk only	8 (24.2%)	13 (9.5%)	10 (10.6%)	6 (11.8%)
Both breast and formula (bottle) milk	3 (9.1%)	5 (3.6%)	2 (2.1%)	2 (3.9%)
Unsure	0	8 (5.8%)	3 (3.2%)	2 (3.9%)
Feeding outcomes				
**Initiated breastfeeding**				
% who intended to exclusively breastfeed	21/22 (95.5%)	96/97 (99.0%)	67/67 (100%)	39/39 (100%)
% who intended some breastfeeding or unsure	21/25 (84.0%)	103/109 (94.5%)	68/71 (95.8%)	41/43 (95.3%)
**On discharge from hospital / labour care**				
Exclusive breastfeeding (% of those who intended exclusive breastfeeding)	18/22 (81.8%)	74/90 (82.2%)	53/60 (88.3%)	33/39 (84.6%)
Some breastfeeding (% of those who intended some or unsure)	21/25 (84.0%)	88/101 (87.1%)	57/63 (90.5%)	37/43 (86.0%)
**On discharge from maternity care**				
Exclusive breastfeeding (% of those who intended exclusive breastfeeding)	15/22 (68.2%)	62/81 (76.5%)	47/55 (85.5%)	23/35 (65.7%)
Some breastfeeding (% of those who intended some or unsure)	18/23 (78.3%)	76/89 (85.4%)	50/55 (90.9%)	28/37 (75.7%)

**Table:** 

Table 14 Legend: Terms, Abbreviations and How/When data was collected	
*Denominator*	This is the total number of cases where we have data available. The percentage rate is calculated by dividing the number of outcomes by the number of cases available (denominator).

### Fidelity outcomes

**Table 15 Key Questions:**

1) Service leaders: How does the presence of an OptiBreech team member affect adherence to the OptiBreech Algorithm?
2) Service users: How does the presence of an OptiBreech team member affect my ability to give birth in an upright position if I want to? How does the presence of an OptiBreech team member affect the likelihood that my baby’s umbilical cord will remain intact until after s/he has started breathing?

**Table 15.** Fidelity criteria, by presence of an OptiBreech team member.

Fidelity Criteria	OptiBreech team member who fulfilled all proficiency criteria present	OptiBreech team member present who had completed OptiBreech training package	No OptiBreech trained professional present
N (%) – *denominator listed when data* * are missing*	30/39 (76.9%)	34/39 (87.2%)	5/39 (12.8%)
Profession of lead attendant			
midwife	26/30 (86.7%)	28/34 (82.4%)	3/4 (75%)
obstetrician	4/30 (13.3%)	6/34 (17.6%)	1/4 (25.0%)
Maternal birth position			
upright	23/30 (76.7%)	25/34 (73.5%)	1/5 (20.0%)
supine or sitting on bed	2/30 (6.7%)	3/34 (8.8%)	2/5 (40.0%)
lithotomy	5/30 (16.7%)	6/34 (17.6%)	2/5 (40.0%)
Algorithm timings			
≤3 minutes pelvis-to-birth	16/22 (72.7%)	18/25 (72.0%)	2/2 (100%)
≤5 minutes pelvis-to-birth	19/22 (86.4%)	22/25 (88.0%)	5/5 (100%)
≤7 minutes rumping-to-birth	26/27 (96.3%)	30/31 (96.8%)	4/4 (100%)
Neonatal transition			
Inflation breaths initiated	8/30 (26.7%)	9/34 (26.5%)	2/5 (40.0%)
Umbilicus intact until after onset of respirations	17/30 (56.7%)	19/34 (55.9%)	2/5 (40.0%)
Umbilicus intact until after onset of respirations when inflation breaths NOT initiated	16/22 (72.7%)	18/25 (72.0%)	2/3 (66.7%)
Umbilicus intact until after onset of respirations when inflation breaths initiated	1/8 (12.5%)	1/9 (11.1%)	0/2

**Table:** 

Table 15 Legend: Terms, Abbreviations and How/When data was collected	
*Denominator*	This is the total number of cases where we have data available. The percentage rate is calculated by dividing the number of outcomes by the number of cases available (denominator).
*Upright*	Upright maternal birth positions include kneeling, hands and knees, standing and squatting positions.
*Supine*	Supine maternal birth positions include positions where the mother is positioned on her back or in a reclined sitting position.
*Lithotomy*	Lithotomy birthing position is when the mother is positioned on her back, with legs held up and open in stirrups.
*Umbilicus*	The baby’s umbilical cord. The time of clamping and cutting is recorded in birth records.
*Intact*	Not clamped or cut.
*Respirations*	Baby’s breathing. The time the baby begins breathing on their own is recorded in birth records.
*Inflation breaths*	If a baby is born in poor condition and attendants decide that they need assistance, the first step in neonatal resuscitation is called ‘inflation breaths.’ These are long, slow puffs of air to inflate the lungs and help remove any remaining amniotic fluid in the lungs.

**Table 16 Key Questions:**

1) Service leaders: How does maintaining the umbilicus intact until after the onset of respirations impact neonatal outcomes?
2) Service users: Will keeping the umbilicus intact until after my baby has started breathing affect whether we need to be separated due to admission to the neonatal unit?

**Table 16.** Incidence of neonatal admission, analysed by whether the umbilicus remained intact.

	Umbilicus intact until after the onset of respirations	
	Yes	No
N (%) – *denominator listed when* * data are missing*	111/169 (65.7%)	58/169 (34.3%)
**Apgar under 7 at 5 minutes**		
no	109/109 (100%)	55/57 (96.5%)
yes	0	2/57 (3.5%)
**Admission to transitional care**		
no	104/111 (93.7%)	46/58 (79.3%)
yes	7/111 (6.3%)	12/58 (20.7%)
**Admission to NICU/SCBU**		
no	109/111 (98.2%)	53/58 (91.4%)
yes	2/111 (1.8%)	5/58 (8.6%)

**Table:** 

Table 16 Legend: Terms, Abbreviations and How/When data was collected	
*Denominator*	This is the total number of cases where we have data available. The percentage rate is calculated by dividing the number of outcomes by the number of cases available (denominator).
*Apgar*	A measure of the physical condition of a newborn infant. It is obtained by adding points (2, 1 or 0) for heart rate, respiratory effort, muscle tone, response to stimulation, and skin coloration. It is measured on a scale of 0–10, where 10 represents the best possible condition. It is measured at 1, 5 and 10 minutes after birth.
*Transitional care*	Neonatal transitional care supports hospital-resident mothers as primary care providers for their babies with care requirements in excess of normal newborn care, but who do not require to be in a neonatal unit. In this report, admission is measured at the time of discharge from birth care.
*NICU*	Neonatal intensive care unit. This is for babies who need the highest level of medical and nursing support. In this report, admission is measured at the time of discharge from birth care.
*SCBU*	Special care baby unit. This is a neonatal unit for babies who do not need intensive care. In this report, admission is measured at the time of discharge from birth care.
*Umbilicus*	The baby’s umbilical cord. The time of clamping and cutting is recorded in birth records.
*Intact*	Not clamped or cut.
*Respirations*	Baby’s breathing. The time the baby begins breathing on their own is recorded in birth records.
*Inflation breaths*	If a baby is born in poor condition and attendants decide that they need assistance, the first step in neonatal resuscitation is called ‘inflation breaths.’ These are long, slow puffs of air to inflate the lungs and help remove any remaining amniotic fluid in the lungs.

## Discussion

Data available as of 8 September 2023 indicate that providing support for planned VBB within an OptiBreech collaborative care pathway has been as safe as a planned cephalic birth in the same hospitals. While this is the largest data set of planned vaginal breech births published in the UK since 2005 ^29^, the sample size is still too small to evaluate rare but important outcomes, such as severe neonatal morbidity or mortality. The Royal College of Obstetricians and Gynaecologists (RCOG) guideline estimates that perinatal mortality following planned caesarean birth at 39 weeks is 0.5/1000, following planned cephalic birth is 1/1000, and following planned vaginal breech birth is 2/1000 ^5^. Evaluating OptiBreech care for this outcome will require thousands of births. Our intention with the multiple trials cohort is to facilitate multiple nested randomised controlled trials to answer important questions about breech care with different endpoints, while accumulating a sufficient sample to evaluate rare outcomes such as perinatal mortality. This will refine the OptiBreech care pathway for maximum efficiency and effectiveness, for all women requiring breech care.

Prior to the start of this cohort, two preliminary studies were done. The first evaluated the OptiBreech training package (‘Physiological Breech Birth’) within NHS settings ^15^. This observed a serious neonatal morbidity rate of 0/21 (same composite, 0%) among births attended by an OptiBreech trained attendant, compared to 5/69 (7.2%) among births where the attendant had NOT attended the training ^15^. This suggests the results of the Term Breech Trial are still relevant to standard care within the UK ^30^.

The second evaluated the feasibility of implementing OptiBreech collaborative team care for planned VBBs ^18^. In this study, among 82 planned VBBs, one serious adverse outcome (same composite, 1.2%) occurred. In the OptiBreech cohort reported in this paper, 97 women have planned a VBB, with no serious adverse neonatal outcomes. We have therefore reported 200 prospective VBBs across three studies, with one serious adverse neonatal outcome (0.5%). This is very near to the rate of adverse outcomes observed among low-risk women planning cephalic (head-first) births in the UK-based Birthplace in England study (0.43%) ^31^. Neonatal admission rates across the feasibility studies following planned VBBs have been below 5%, similar to rates for all term births in the UK ^32^.

We feel our results so far are due to strong qualitative work to develop the programme theory for the OptiBreech collaborative care model. Our logic model was developed and refined through: frequent and meaningful PPIE activities; systematic reviews to establish background, questions and women’s experiences ^8, 22, 33^; Delphi consensus methods, including clinicians and service users, on core competencies and important outcomes ^4, 16, 24, 25, 34^; grounded theory methods to describe how clinicians learn breech skills ^2, 3^; feasibility work to prepare for a substantive clinical trial ^1, 18^; and a pilot randomised trial ^28^.

Detailed observational work also theoretically underpins our practice guidelines, including video analysis and case control studies to define ranges of ‘normal’ in breech births ^8– 10^. The OptiBreech Algorithm aims to reduce the leading cause of breech birth-related injury: asphyxia ^8– 10^. Our team has raised concerns previously that current guidelines are not optimally safe ^10, 35^. Historically, breech practice has not been based on evidence, particularly around the expected time intervals as the breech baby emerges ^8^. This is the fourth paper in which we report that, in most cases with good outcomes, the birth has completed *within* three to five minutes of the birth of the fetal pelvis ^9, 10, 18^. We strongly feel this should be regarded as ‘normal for breech.’

Our OptiBreech guideline continues to recommend attendants aim for the birth to be complete *within* five minutes from the birth of the pelvis, including time for manoeuvres. It is especially important for novices, who are inherently less confident to intervene, to have clear guidelines that alert them when a threshold of increased risk is approaching. Current RCOG ^5^ and PROMPT ^36^ guidelines recommend assisting only *after* five minutes have passed following birth of the pelvis and emphasise a ‘hands off’ approach. Our guideline promotes using maternal effort and movement (‘wiggle and push’) as a first-line intervention if advancement pauses for 30 seconds or more at any point after the birth of the pelvis. This optimises maternal agency and minimises the need for attendants to manually intervene. We feel waiting five minutes to assist the birth offers no advantages. Rather, it increases the risk of an adverse outcome should the attendant discover after five minutes that the delay is due to arm or head entrapment, which may take significant time to resolve.

There are limitations to presenting interim data in this way. We cannot assume that the results will continue in the same manner, and a much larger sample size will be needed to determine overall safety. Although local investigators have an obligation to report serious adverse outcomes, currently missing data may reveal in future analyses outcomes that are less positive than they currently appear. These results also do not apply to all planned VBBs. The OptiBreech teams follow a specific care algorithm ^10^ with manoeuvres specific to upright breech birth ^37^, the presence of an OptiBreech team member increases the likelihood that this will be followed ^2, 18^, and their presence facilitates shared learning from each birth throughout the team. Outside of this model of care, the absence of one or more of these potential mechanisms may impact outcomes.

This analysis has also helped us to identify areas that require further support for cultural change. Our guideline also recommends that, should the baby be born in poor condition, resuscitation be initiated with the umbilical cord intact ^16^. Current evidence supports leaving the umbilical cord intact until after the onset of respirations ^38, 39^, and service users have identified this in PPIE work as an important and under-studied outcome for them ^25, 40^. However, these data indicate OptiBreech teams are not currently achieving this. Understanding why and developing a strategy for addressing this difficult area of implementation will require further research and collaboration with neonatal teams. While most research has focused on premature babies ^41^, for whom this practice provides significant benefit, the difference in neonatal admission rates in our data suggest it may be beneficial for term babies as well.

We remind readers that this is an interim data set. Some data points are missing, and in a small data set, results can change dramatically in a short period. However, at this point, we are encouraged by this very positive set of pilot data, which will grow with each trial funded.

## Data Availability

Figshare: OptiBreech Care IRAS 303028 Data Set, https://doi.org/10.6084/m9.figshare.c.6386370.v2 ^42^. This project contains the following underlying data: OptiBreech Care IRAS 303028 cohort interim data to 8 September 2023 OptiBreech Codebook data to 8 September 2023 STROBE and GRIPP2 checklists for analysis of OptiBreech Care data to 8 September 2023 Data are available under the terms of the Creative Commons Attribution 4.0 International license (CC-BY 4.0).
