# Supplementary material for: How safe is it to plan a vaginal breech birth with OptiBreech collaborative care?: analysis of cumulative data within the OptiBreech Multiple Trials Cohort
Source: NIHR Open Res. 2023 Nov 23;3:61. [Version 1] doi: 10.3310/nihropenres.13500.1 (PMC12867103; doi:10.3310/nihropenres.13500.1)
Supplement: Peer review history

# Reviewer report: Reviewer response for version 1

**DOI:** 10.3310/nihropenres.14650.r31049

**Published:** 2024-04-08

**Authors:** Robyn Schafer

**Affiliations:** 1 School of Nursing, Division of Advanced Practice, Rutgers University Newark, Newark, New Jersey, USA; 2 Robert Wood Johnson Medical School, Department of OBGYN & Reproductive Sciences, Rutgers University Newark, New Brunswick, New Jersey, USA

Thank you for the invitation to review this original research article entitled, “How safe is it to plan a vaginal breech birth with OptiBreech collaborative care?” This publication provides an interim analysis of maternal and neonatal outcomes in a prospective trial of women planning a vaginal breech birth who received either standard NHS or OptiBreech care (including both randomized and non-randomized cohorts). Although the sample size is too small to detect meaningful differences in rare outcomes, the descriptive data are useful to inform future research efforts and guide decision-making regarding integration of this care model into the health system.

 The research topic is timely and important, and the study methodology is appropriate to the research questions and thoroughly described. Suggestions for increased detail and clarity to the methods/analysis to ensure reproducibility are provided below. Ethical standards for consent and participant involvement have been met. Equity analyses were included to assess accessibility and differences in outcomes for persons from minoritized groups.

 The authors should be commended on undertaking this important research effort to ensure that pregnant and birthing people with a breech fetus have options for high-quality care and informed choice for mode of birth.

 The following suggestions and questions might be considered to guide revisions and further strengthen this manuscript:

**Introduction**

- *Use of gendered language*. This publication is inconsistent in its use of gendered (i.e., “women” and “mothers”) and gender-inclusive terminology (i.e., “birthing people”). Furthermore, the authors state that “recruitment focused on women….” Does this mean that pregnant individuals who did not identify as women were intentionally not recruited? I would suggest the authors consider the use of gendered and gender-inclusive terminology throughout to accurately reflect the study protocol and, to the fullest extent possible, be inclusive with persons with diverse gender identities.
- *Confusing/unclear statements. *(1) The authors state, “Balancing these risks [of surgical delivery and birth trauma] through person-centred care, centred on the person’s values and life context, is improved when obstetricians and midwives have skill and experience in vaginal breech birth (VBB).” Although I wholeheartedly agree in the importance of person-centered care for vaginal breech birth, it is unclear to me how the incorporation of PCC will improve the balance of surgical risks and trauma based on provider experience. Further explanation would be helpful. (2) The authors state, “Current NHS strategies to reduce risk focus on reducing the numbers of VBBs, through external cephalic version (ECV, turning the baby head-down) and/or planned caesarean birth (CB). An unintended effect has been decline in health care professionals’ skills.” I assume by “health professionals’ skills,” the authors mean those skills specific to vaginal breech birth? Such policies would logically increase providers’ skills in ECV, so further clarification would be useful. (3) The authors state, “This strategy has reduced the VBBs we can anticipate, resulting in a lack of learning opportunities for staff to safely manage those we cannot predict.” Do the authors mean that this strategy has reduced the number of vaginal breech births planned antenatally? I would hope that skilled birth attendants would be able to determine fetal position in labor prior to onset of second stage so that most vaginal breech births would be anticipated/predicted in the intrapartum setting. This statement is especially confusing given that the OptiBreech model includes those breech presentations discovered during labor.

**Study protocol**

 Additional information recommended to include:

- This publication would benefit from inclusion of a clear definition of “standard care” and the ways in which OptiBreech care is different to orient the reader who may not be familiar with UK NHS and OptiBreech standards.
- I would encourage the study authors to provide more specific exclusion criteria regarding “absolute reason for caesarean section” to list all relevant conditions that warranted exclusion from the study cohort.
- There is no mention of labor induction or augmentation. The authors might consider adding information about labor management guidelines and the use of induction or augmentation across cohorts.
- The manuscript states, “Generally, they were offered ECV as a first-line intervention …” Although Table 5 presents data regarding ECV, it is unclear how many participants were offered ECV, rationale for those that were not offered, what percent attempted the procedure, and rates of success in resulting in cephalic presentation at onset of labor.
- The manuscript states that “professionals with advanced training in physiological breech birth attend VBBs whenever possible.” The lack of a clinician experienced in vaginal breech birth at all births in OptiBreech care is concerning given that best available evidence and consensus clinical practice recommendations specify the requirement of an experienced clinician for optimal health outcomes and, as this manuscript notes, there are wide variations in provider experience with regard to breech birth. It is unclear if the authors are implying by “advanced training” that clinicians had completed OptiBreech training or if other forms of breech skills training would qualify. Additional information about the education/training and level of experience of attendants should be described in greater detail.

**Results**

- The authors have reported outcomes based on type of breech presentation as either (a) extended/frank or (b) any other or uncertain. To provide more meaningful comparison, I would suggest removing “uncertain” presentations from the “any other” group to create a third category, since “uncertain” could also include extended/frank presentations.
- The authors list both “vaginal breech birth” and “forceps breech birth,” but it is unclear if forceps is a subset of vaginal breech birth or if the former category includes only spontaneous vaginal births.
- Could rationale for in-labour and pre-labour caesarean births be provided?
- It appears the authors have used 3 cm dilation as the determining factor for differentiating “early” v. “active” labor. This is not consistent with standard definitions of active labor and should be explained.
- Rates of episiotomy in OptiBreech care groups are quite high. Could the authors expound on the use of episiotomy in this model of care or provide rationale for the difference in episiotomy rates between cohorts?

**Discussion**

- The discussion section could be strengthened by including implications for patient education/counseling and recommendations for health policy aligned with the research questions.

Thank you for the opportunity to review this important research.

Is the work clearly and accurately presented and does it cite the current literature?

Yes

If applicable, is the statistical analysis and its interpretation appropriate?

Yes

Are all the source data underlying the results available to ensure full reproducibility?

Yes

Is the study design appropriate and is the work technically sound?

Yes

Are the conclusions drawn adequately supported by the results?

Yes

Are sufficient details of methods and analysis provided to allow replication by others?

Partly

Reviewer Expertise:

Midwifery, perinatal and reproductive health, clinical decision-making, evidence-based practice, person-centered care, qualitative and mixed methods research

I confirm that I have read this submission and believe that I have an appropriate level of expertise to confirm that it is of an acceptable scientific standard, however I have significant reservations, as outlined above.

# Response

**Published:** 2024-12-03

**Authors:** Shawn Walker

**Affiliations:** Women and Children's Health, King's College London, London, UK

Thank you for the invitation to review this original research article entitled, “How safe is it to plan a vaginal breech birth with OptiBreech collaborative care?” This publication provides an interim analysis of maternal and neonatal outcomes in a prospective trial of women planning a vaginal breech birth who received either standard NHS or OptiBreech care (including both randomized and non-randomized cohorts). Although the sample size is too small to detect meaningful differences in rare outcomes, the descriptive data are useful to inform future research efforts and guide decision-making regarding integration of this care model into the health system.

 The research topic is timely and important, and the study methodology is appropriate to the research questions and thoroughly described. Suggestions for increased detail and clarity to the methods/analysis to ensure reproducibility are provided below. Ethical standards for consent and participant involvement have been met. Equity analyses were included to assess accessibility and differences in outcomes for persons from minoritized groups.

 The authors should be commended on undertaking this important research effort to ensure that pregnant and birthing people with a breech fetus have options for high-quality care and informed choice for mode of birth.

 The following suggestions and questions might be considered to guide revisions and further strengthen this manuscript:

**Introduction**

- *Use of gendered language*. This publication is inconsistent in its use of gendered (i.e., “women” and “mothers”) and gender-inclusive terminology (i.e., “birthing people”). Furthermore, the authors state that “recruitment focused on women….” Does this mean that pregnant individuals who did not identify as women were intentionally not recruited? I would suggest the authors consider the use of gendered and gender-inclusive terminology throughout to accurately reflect the study protocol and, to the fullest extent possible, be inclusive with persons with diverse gender identities.

**We have added a clarifying statement. As a general rule throughout the OptiBreech research, after working with our PPIE group, we use sex-specific terminology and acknowledge the need to be inclusive of gender-diverse individuals as well. We have tried to strike a balance of signalling our desire to respect diverse gender identities, while also being clear and concise.**

  

- *Confusing/unclear statements. *(1) The authors state, “Balancing these risks [of surgical delivery and birth trauma] through person-centred care, centred on the person’s values and life context, is improved when obstetricians and midwives have skill and experience in vaginal breech birth (VBB).” Although I wholeheartedly agree in the importance of person-centered care for vaginal breech birth, it is unclear to me how the incorporation of PCC will improve the balance of surgical risks and trauma based on provider experience. Further explanation would be helpful.

**Further explanation has been added. Providers cannot offer the choice of a VBBs if they do not have the skills to do so safety, but due to the way services are currently delivered in standard services, experience in VBB is difficult to acquire.**

  

- (2) The authors state, “Current NHS strategies to reduce risk focus on reducing the numbers of VBBs, through external cephalic version (ECV, turning the baby head-down) and/or planned caesarean birth (CB). An unintended effect has been decline in health care professionals’ skills.” I assume by “health professionals’ skills,” the authors mean those skills specific to vaginal breech birth? Such policies would logically increase providers’ skills in ECV, so further clarification would be useful.

**VBB added for clarity.**

  

- (3) The authors state, “This strategy has reduced the VBBs we can anticipate, resulting in a lack of learning opportunities for staff to safely manage those we cannot predict.” Do the authors mean that this strategy has reduced the number of vaginal breech births planned antenatally? I would hope that skilled birth attendants would be able to determine fetal position in labor prior to onset of second stage so that most vaginal breech births would be anticipated/predicted in the intrapartum setting. This statement is especially confusing given that the OptiBreech model includes those breech presentations discovered during labor.

**Unfortunately, all available studies on the accuracy of palpation indicate that it is not consistently accurate, even with highly experienced attendants. Without universal ultrasound scans, the rate of diagnosis in labour is about 20%. Even with universal ultrasound, unstable lies mean the occasional breech presentation will be discovered in labour, and a small number of fetuses will turn in labour.**

**Study protocol**

 Additional information recommended to include:

- This publication would benefit from inclusion of a clear definition of “standard care” and the ways in which OptiBreech care is different to orient the reader who may not be familiar with UK NHS and OptiBreech standards.

**We have clarified that our description of OptiBreech care is based on the ways it differs from standard care.**

- I would encourage the study authors to provide more specific exclusion criteria regarding “absolute reason for caesarean section” to list all relevant conditions that warranted exclusion from the study cohort.

**Thank you for this suggestion. The exclusion criteria are replicated here verbatim from the protocol. We acknowledge there may be differences in perception around what constitutes an absolute reason for caesarean section and will consider this for the future.**

  

- There is no mention of labor induction or augmentation. The authors might consider adding information about labor management guidelines and the use of induction or augmentation across cohorts.

**We do not currently have a guideline on this, and practice varied from site to site. The nature of the intervention – an experienced team – meant that some more experienced team were comfortable with this, and some were not. We are currently conducting a systematic review on outcomes for induced breech labours, and we hope to report the small amount of data we have where this occurred separately, in preparation for a study on this specific topic.**

  

- The manuscript states, “Generally, they were offered ECV as a first-line intervention …” Although Table 5 presents data regarding ECV, it is unclear how many participants were offered ECV, rationale for those that were not offered, what percent attempted the procedure, and rates of success in resulting in cephalic presentation at onset of labor.

**While we can understand the interest in this data, our focus in this study was on the offer of VBB and its outcomes. These were not among the outcome we prospectively aimed to report.**

  

- The manuscript states that “professionals with advanced training in physiological breech birth attend VBBs whenever possible.” The lack of a clinician experienced in vaginal breech birth at all births in OptiBreech care is concerning given that best available evidence and consensus clinical practice recommendations specify the requirement of an experienced clinician for optimal health outcomes and, as this manuscript notes, there are wide variations in provider experience with regard to breech birth.

**This is correct – the evidence indicates that an experienced clinician improves outcomes. This is one of the first studies to use objective criteria to determine ‘experienced,’ based on previous consensus research. One of the main barriers to offering women the choice of VBB is the inconsistency with which an experienced provider can be in attendance, and the inability to guarantee their presence. Despite our best efforts, due to the unpredictability of labour, we were not able to guarantee this either – for example, at least one precipitous labour occurred with no attendant at home. What we can do is to offer women information on the likelihood that an attendant with training and experience would be in attendance, so that they can make an informed choice about whether to proceed.**

**In this revision, we have reported the experience levels of attendants as recorded.**

  

-  It is unclear if the authors are implying by “advanced training” that clinicians had completed OptiBreech training or if other forms of breech skills training would qualify. Additional information about the education/training and level of experience of attendants should be described in greater detail.

**In this sentence, we reference the evaluations of the physiological breech birth training programme attendants had completed. The syllabus for the evaluated training is included in those publications, for replication purposes.**

**Experience levels are reported in this revision.**

**Results**

- The authors have reported outcomes based on type of breech presentation as either (a) extended/frank or (b) any other or uncertain. To provide more meaningful comparison, I would suggest removing “uncertain” presentations from the “any other” group to create a third category, since “uncertain” could also include extended/frank presentations.

**Our study was designed with extended or non-extended/flexed as the two minimisation categories. When we discovered that occasionally the type of breech was not identified, we conferred with our PIs about how to handle this, and this was the conclusion that was drawn. Uncertain presentations were therefore not reported separately and cannot be with the data we hold.**

  

- The authors list both “vaginal breech birth” and “forceps breech birth,” but it is unclear if forceps is a subset of vaginal breech birth or if the former category includes only spontaneous vaginal births.

**Further clarification added in keys below tables.**

  

- Could rationale for in-labour and pre-labour caesarean births be provided?

**We have incorporated the latest guidance on describing types of birth, based on the Re:Birth project, led by the RCM. https://rcm.org.uk/rebirth/**

**We have clarified in the key tables what each phrase includes, for clarity.**

  

- It appears the authors have used 3 cm dilation as the determining factor for differentiating “early” v. “active” labor. This is not consistent with standard definitions of active labor and should be explained.

**We have now included a justification, which is that we used the same categories as those used in the Term Breech Trial for consistency. All births included in these categories were vaginal births, despite a caesarean birth being initially planned, suggesting that they were indeed in active labour when diagnosed.**

****

  

- Rates of episiotomy in OptiBreech care groups are quite high. Could the authors expound on the use of episiotomy in this model of care or provide rationale for the difference in episiotomy rates between cohorts?

**In this publication, we have focused on reporting the outcomes. There are many details that need more time and space to unpick and examine more closely. We agree, this is one of these. The higher rate of episiotomy compared to cephalic births is consistent with all other studies that report perineal outcomes for both cohorts.**

**Discussion**

- The discussion section could be strengthened by including implications for patient education/counseling and recommendations for health policy aligned with the research questions.

**As this was pilot/feasibility work, we are reluctant to make premature recommendations for counselling and policy changes. Our first priority is to obtain the funding to conduct a substantive study that would credibly result in data to inform policy decisions.**

**Thank you for the opportunity to review this important research.**

**Thank you for your thoughtful and considered review, and your kind words.**

# Reviewer report: Reviewer response for version 1

**DOI:** 10.3310/nihropenres.14650.r31389

**Published:** 2024-04-01

**Authors:** Jennifer Barrowclough

**Affiliations:** 1 Midwifery Department, Auckland University of Technology, Auckland, New Zealand

Congratulations on conducting the OptiBreech collaborative care study and documenting the comprehensive interim analysis that may lead to improved outcomes for women and their babies following breech presentation. I suggest some minor revisions and points to consider below. The manuscript is well written but due to the multi-dimensional cohort, the reader may need extra clarification of these where relevant throughout the text. There appears to be some formatting issues for some words that include unnecessary spaces and dashes. More importantly there appear to be some errors in the tables and need for some data concerning adverse events to be presented in separate rows. 

**Comments**

**1. Abstract:**

 Results

 It would be helpful to insert '(Table 8)', after first sentence of Results in the Abstract. It would help clarify the results if you insert the text '(all presentations)' after 'achieved a vaginal birth' in line one of Results.

 Please correct percentage 2/52 (3.7%) in Results - it should be 3.8%.

 Please clarify '...planned a cephalic birth'. Does this refer to women who initially had a breech presentation which later turned? Is so, consider inserting the words 'after breech turned' after the words 'cephalic birth.', if this is correct.

**2. Methods:**

**Procedures**

 First paragraph- Please insert 'ECV' after 'declined' i.e. '...regarding mode of birth if declined [ECV].'

**Outcomes**

 First paragraph - I suggest you insert ‘outcomes including:...’ after ‘short term’

 Please insert  '1-16' after 'Below each table'

**Statistical analysis**

 In paragraph 5, please explain by what means a fetus was 'discovered' to be presenting breech in labour...' i.e. was this by confirmed by scan?

 Paragraph 6, Should PPI be PPIE? Please correct as needed, here and subsequently.

 Paragraph 6, clause 6), Perhaps rephrase as 'our PPI group was keen to ensure participants were not disadvantaged by nationality, ethnicity or skin colour’? and later consider using the term 'non-Caucasian British' or 'non-Caucasian-non British' in the tables.  Was this a unanimous decision or a proportion of the PPI group? How many members were in the PPI group? Reference to skin colour in academic publications is not current practice, though I appreciate your point and Lancet citation with evidence of inequity based on skin colour. If you still intend to refer to colour, you may need to emphasis more why you are doing this, and use footnote for Table 1 to provide the Lancet citation after the first two rows under the heading 'Any other ethnic group'.

**3. Results:**

 The Key Questions that are addressed in each table is a novel and useful way to present the findings. However, the Results section could benefit with a summary of the findings similar to in your abstract, as it otherwise goes straight to the tables which are numerous.

**Tables**

 Please capitalise the first letter of the first word for all outcomes throughout all tables.

 Table 1, I suggest you rephrase the heading 'Diagnosis prior to labour', to ‘Timing of diagnosis’, given row 2 under this heading refers to 'in labour...'

 Table 3, There should be a separate row for mortality.  Please clarify why the legend describing the definition of severe morbidity, does not appear to be reflected in the numbers (%) provided for 'Severe neonatal morbidity/mortality prior to discharge.' - Please make it possible for the reader to see the numbers for the parts that make up this composite outcome.

 Please check all percentages in all tables.

**Discussion:**

 The Discussion refers to relevant studies which align with the design of OptiBreech cohort study, and considerations for the OptiBreech guideline.

 Paragraph 3, the sentence 'We have therefore reported 200 prospective VBBs across three studies, with one serious adverse neonatal outcomes (0.5%)', is confusing. Please insert 'including the present study' after 'three studies' to improve clarity. (if this is what is meant).

 Second to last paragraph, first sentence, please explain what you mean by cultural change? Are you referring to the culture of labour care concerning the umbilical cord or other cultural changes?

 Thank you for undertaking and continuing this most important work, and your preparatory studies.

Is the work clearly and accurately presented and does it cite the current literature?

Partly

If applicable, is the statistical analysis and its interpretation appropriate?

Yes

Are all the source data underlying the results available to ensure full reproducibility?

Partly

Is the study design appropriate and is the work technically sound?

Yes

Are the conclusions drawn adequately supported by the results?

Yes

Are sufficient details of methods and analysis provided to allow replication by others?

Yes

Reviewer Expertise:

Fetal malposition; Use of maternal posture to improve health outcomes of fetal malposition for mothers and their babies.

I confirm that I have read this submission and believe that I have an appropriate level of expertise to confirm that it is of an acceptable scientific standard, however I have significant reservations, as outlined above.

# Response

**Published:** 2024-12-03

**Authors:** Shawn Walker

**Affiliations:** Women and Children's Health, King's College London, London, UK

Congratulations on conducting the OptiBreech collaborative care study and documenting the comprehensive interim analysis that may lead to improved outcomes for women and their babies following breech presentation. I suggest some minor revisions and points to consider below. The manuscript is well written but due to the multi-dimensional cohort, the reader may need extra clarification of these where relevant throughout the text. There appears to be some formatting issues for some words that include unnecessary spaces and dashes. More importantly there appear to be some errors in the tables and need for some data concerning adverse events to be presented in separate rows. 

**Comments**

**1. Abstract:**

 Results

 It would be helpful to insert '(Table 8)', after first sentence of Results in the Abstract. It would help clarify the results if you insert the text '(all presentations)' after 'achieved a vaginal birth' in line one of Results.

**Author Response: **Referring to a Table in an Abstract is not standard practice, so we have not done this. We feel that (all presentations) would be confusing in the Abstract without the benefit of further explanation.

 Please correct percentage 2/52 (3.7%) in Results - it should be 3.8%.

**Author Response: **All results have been updated with the remaining data and double-checked.

 Please clarify '...planned a cephalic birth'. Does this refer to women who initially had a breech presentation which later turned? Is so, consider inserting the words 'after breech turned' after the words 'cephalic birth.', if this is correct.

**Author Response: **We have added ‘after spontaneous version or external cephalic version’ for clarity

**2. Methods:**

**Procedures**

 First paragraph- Please insert 'ECV' after 'declined' i.e. '...regarding mode of birth if declined [ECV].'

**Author Response: **We have changed this to ‘if ECV failed or was declined’ for clarity.

**Outcomes**

 First paragraph - I suggest you insert ‘outcomes including:...’ after ‘short term’

 Please insert  '1-16' after 'Below each table'

**Author Response: **Changed as suggested.

**Statistical analysis**

 In paragraph 5, please explain by what means a fetus was 'discovered' to be presenting breech in labour...' i.e. was this by confirmed by scan?

**Author Response: **The word ‘discovered’ is one of the Inclusion criteria from the protocol, so cannot be altered. This discovery may be by ultrasound scan if time permits, but sometimes it is determined solely by vaginal examination or visualisation of the buttocks on the perineum.

 Paragraph 6, Should PPI be PPIE? Please correct as needed, here and subsequently.

**Author Response: **We have searched for all instances of PPI and replaced with PPIE.

 Paragraph 6, clause 6), Perhaps rephrase as 'our PPI group was keen to ensure participants were not disadvantaged by nationality, ethnicity or skin colour’? and later consider using the term 'non-Caucasian British' or 'non-Caucasian-non British' in the tables.  Was this a unanimous decision or a proportion of the PPI group? How many members were in the PPI group? Reference to skin colour in academic publications is not current practice, though I appreciate your point and Lancet citation with evidence of inequity based on skin colour. If you still intend to refer to colour, you may need to emphasis more why you are doing this, and use footnote for Table 1 to provide the Lancet citation after the first two rows under the heading 'Any other ethnic group'.

**Author Response: **I cannot remember how many people were present when this was discussed, but the specific language used was suggested by a black member of research group, based on diversity reporting guidelines used in the UK. The group were unanimous that we needed to get over our discomfort of referring to skin colour and make it visible. We have added a reference to the Lancet article as suggested in the text.

 We have also added a further explanatory paragraph in the discussion.

**3. Results:**

 The Key Questions that are addressed in each table is a novel and useful way to present the findings. However, the Results section could benefit with a summary of the findings similar to in your abstract, as it otherwise goes straight to the tables which are numerous.

**Tables**

 Please capitalise the first letter of the first word for all outcomes throughout all tables.

 Table 1, I suggest you rephrase the heading 'Diagnosis prior to labour', to ‘Timing of diagnosis’, given row 2 under this heading refers to 'in labour...'

**Author Response: **Changed as suggested.

 Table 3, There should be a separate row for mortality. 

**Author Response: **This has been added to all neonatal outcome tables.

 Please clarify why the legend describing the definition of severe morbidity, does not appear to be reflected in the numbers (%) provided for 'Severe neonatal morbidity/mortality prior to discharge.' - Please make it possible for the reader to see the numbers for the parts that make up this composite outcome.

**Author Response: **This has been added to all neonatal outcome tables.

 Please check all percentages in all tables.

**Author Response: **These have been checked.

**Discussion:**

 The Discussion refers to relevant studies which align with the design of OptiBreech cohort study, and considerations for the OptiBreech guideline.

 Paragraph 3, the sentence 'We have therefore reported 200 prospective VBBs across three studies, with one serious adverse neonatal outcomes (0.5%)', is confusing. Please insert 'including the present study' after 'three studies' to improve clarity. (if this is what is meant).

**Author Response: **Changed as suggested.

 Second to last paragraph, first sentence, please explain what you mean by cultural change? Are you referring to the culture of labour care concerning the umbilical cord or other cultural changes?

**Author Response: **We have changed the word ‘cultural’ to ‘clinical’ for clarity.

 Thank you for undertaking and continuing this most important work, and your preparatory studies.

 Thank you for your careful and considered review, and kind words.

# Reviewer report: Reviewer response for version 1

**DOI:** 10.3310/nihropenres.14650.r30827

**Published:** 2023-12-20

**Authors:** Sara Morris

**Affiliations:** 1 Edith Cowan University, Western Australia, Australia

Thank you for the invitation to review the article titled *How safe is it to plan a vaginal breech birth with Optibreech collaborative care?: Analysis of cumulative data within the Optibreech Multiple Trials Cohort*.

 The title of the paper provides a clear outline of the scope of the article which is well written and presented appropriately. The methodological processes are clearly documented and easy to follow, allowing for replication. The authors should be commended for the level of consumer involvement in the co-design of the Optibreech research.  

 The authors have provided adequate justification for the chosen methodology with clearly stated objectives and succinctly reported results demonstrating adequate review of the literature. The use of descriptive statistics is appropriate for this data set.

 Data availability is also outlined within.

 The authors have made appropriate conclusions while acknowledging the limitations on interpretation of the data at this point in the study.  

 The figures and tables presented are clear and numerous. Having corresponding questions connected to the data tables is helpful. Please note that Table 10 contains an asterisk, but it is not included in the Legend.

 The authors should be congratulated on their work.

 I have the following feedback/suggestions/queries:

- I think it would be helpful to separate the data for Category 1 & 2 Caesarean Sections as well as severe morbidity and mortality as there is a significant difference between these categories.

 

- I also think justification is needed for the use of >3cm for being considered active labour when the current literature supports 6cm as established and NHS information sources for consumers reports 4cm as established labour.  

 

- Under the Consent Process section, it states that after the “…pilot trial that recruitment focused on women requesting Optibreech care due to a preference for vaginal birth, regardless of fetal position.” Was this fetal position at the time of admission/birth? Does this mean that women with known cephalic presentations were able to request and obtain Optibreech care or did they experience a breech presentation at some point?

Is the work clearly and accurately presented and does it cite the current literature?

Yes

If applicable, is the statistical analysis and its interpretation appropriate?

Yes

Are all the source data underlying the results available to ensure full reproducibility?

Yes

Is the study design appropriate and is the work technically sound?

Yes

Are the conclusions drawn adequately supported by the results?

Yes

Are sufficient details of methods and analysis provided to allow replication by others?

Yes

Reviewer Expertise:

Women's experiences of breech diagnosis, care and birth & the recommended care for women with breech pregnancies.

I confirm that I have read this submission and believe that I have an appropriate level of expertise to confirm that it is of an acceptable scientific standard.

# Response

**Published:** 2024-12-03

**Authors:** Shawn Walker

**Affiliations:** Women and Children's Health, King's College London, London, UK

Thank you for the invitation to review the article titled *How safe is it to plan a vaginal breech birth with Optibreech collaborative care?: Analysis of cumulative data within the Optibreech Multiple Trials Cohort*.

**Thank you for taking the time to complete such a thorough review, and for your kind words.**

 The title of the paper provides a clear outline of the scope of the article which is well written and presented appropriately. The methodological processes are clearly documented and easy to follow, allowing for replication. The authors should be commended for the level of consumer involvement in the co-design of the Optibreech research.  

 The authors have provided adequate justification for the chosen methodology with clearly stated objectives and succinctly reported results demonstrating adequate review of the literature. The use of descriptive statistics is appropriate for this data set.

 Data availability is also outlined within.

 The authors have made appropriate conclusions while acknowledging the limitations on interpretation of the data at this point in the study.  

 The figures and tables presented are clear and numerous. Having corresponding questions connected to the data tables is helpful. Please note that Table 10 contains an asterisk, but it is not included in the Legend.

**Thank you. This has been removed.**

 The authors should be congratulated on their work.

 I have the following feedback/suggestions/queries:

- I think it would be helpful to separate the data for Category 1 & 2 Caesarean Sections as well as severe morbidity and mortality as there is a significant difference between these categories.

**Thank you for this suggestion. For this report, we focused on the information women and clinicians felt was most significant: how many of the in-labour caesarean births were performed at full dilation. We will bear this in mind for future reports, but we do not feel given the low numbers of births included in this study, further differentiation would add substantial insight.**

  

- I also think justification is needed for the use of >3cm for being considered active labour when the current literature supports 6cm as established and NHS information sources for consumers reports 4cm as established labour.  

**We have now included a justification, which is that we used the same categories as those used in the Term Breech Trial for consistency. All births included in these categories were vaginal births, despite a caesarean birth being initially planned, suggesting that they were indeed in active labour when diagnosed.**

  

- Under the Consent Process section, it states that after the “…pilot trial that recruitment focused on women requesting Optibreech care due to a preference for vaginal birth, regardless of fetal position.” Was this fetal position at the time of admission/birth? Does this mean that women with known cephalic presentations were able to request and obtain Optibreech care or did they experience a breech presentation at some point?

**We have changed this to ‘regardless of whether their baby remained in a breech position’ for clarity.**
